# Unraveling Structure–Performance Relationships in Porphyrin-Sensitized TiO_2_ Photocatalysts

**DOI:** 10.3390/nano13061097

**Published:** 2023-03-18

**Authors:** Belén Vaz, Moisés Pérez-Lorenzo

**Affiliations:** 1 CINBIO, Universidade de Vigo, 36310 Vigo, Spain; 2 Galicia Sur Health Research Institute, 36310 Vigo, Spain

**Keywords:** porphyrins, titanium dioxide, photocatalysis, molecular design

## Abstract

Over the years, porphyrins have arisen as exceptional photosensitizers given their ability to act as chlorophyll-mimicking dyes, thus, transferring energy from the light-collecting areas to the reaction centers, as it happens in natural photosynthesis. For this reason, porphyrin-sensitized TiO_2_-based nanocomposites have been widely exploited in the field of photovoltaics and photocatalysis in order to overcome the well-known limitations of these semiconductors. However, even though both areas of application share some common working principles, the development of solar cells has led the way in what is referred to the continuous improvement of these architectures, particularly regarding the molecular design of these photosynthetic pigments. Yet, those innovations have not been efficiently translated to the field of dye-sensitized photocatalysis. This review aims at filling this gap by performing an in-depth exploration of the most recent advances in the understanding of the role played by the different structural motifs of porphyrins as sensitizers in light-driven TiO_2_-mediated catalysis. With this goal in mind, the chemical transformations, as well as the reaction conditions under which these dyes must operate, are taken in consideration. The conclusions drawn from this comprehensive analysis offer valuable hints for the implementation of novel porphyrin–TiO_2_ composites, which may pave the way toward the fabrication of more efficient photocatalysts.

## 1. Introduction

**Figure 1:**
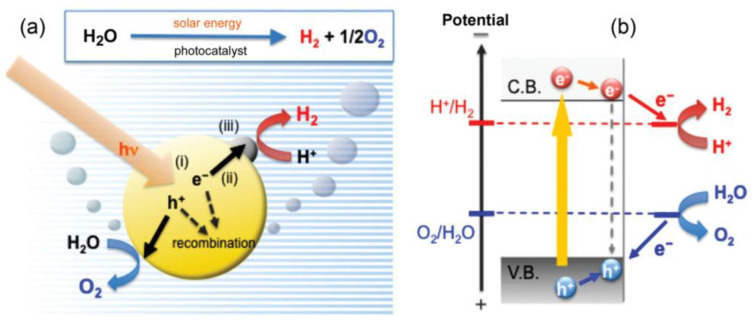
(**a**) Schematic representation of (i) photon absorption, (ii) charge separation and transfer and (iii) surface chemical reactions; (**b**) redox potential requirements for photoinduced water oxidation and reduction processes mediated by a semiconductor photocatalyst. Reproduced from reference [9] with permission from Elsevier, copyright 2010.

Over recent years, the increasing energy demand and contamination effects arising from the continuous usage of fossil fuels have rendered sunlight as a crucial alternative in order to achieve a clean, sustainable and unlimited source of energy. Along these lines, the fact that the full utilization of one hour of solar light would be enough to meet the annual energy demand on Earth [1] has encouraged the search for novel approaches allowing for an efficient conversion and further exploitation of solar energy. It is for this reason that the scientific community is, nowadays, facing the urgent challenge of developing technologies to harvest, transform and efficiently store the sun’s energy into alternative fuels and chemical forms. In this area of research, photocatalysis based on the use of semiconductors has shown great potential, allowing for the realization of significant processes, such as hydrogen production [2], oxygen evolution reactions [3], carbon dioxide reduction [4], wastewater treatments [5] or nitrogen fixation [6], among others. In general, attaining a high-performance photocatalyst involves fulfilling a series of properties so as to promote redox transformations: (1) The bandgap difference between the valence band (VB) and the conduction band (CB) of the semiconductor should be compatible with the energy provided by the absorption of light. In this process, upon illumination, electrons are photoexcited from the VB to the CB, generating the corresponding vacancies (commonly known as holes) and excited electrons in the semiconductor. (2) A good diffusion of charge carriers is vital for an efficient performance and charge utilization, avoiding the recombination of electrons and holes before reaching the surface of the semiconductor material, where the redox reaction takes place. (3) Additionally, the photogenerated electrons and holes must meet the thermodynamic requirements for the final application [7]. Thus, using, as an example, the generation of hydrogen from water, the energy of the CB of the semiconductor must be more negative than the reduction potential for the conversion of water to H_2_ (Figure 1). Likewise, to promote an oxidation reaction such as the oxidation of water to molecular oxygen, the VB of the semiconductor must be more positive than the oxidation potential of water to yield O_2_. In terms of catalytic efficiency, the rate of the catalyzed process may also be greatly influenced by the adsorption capacity and contact surface between the active material and the chemical species to be transformed [8], often determining the overall reaction rate, as in the case of CO_2_ reduction or the photodegradation of organic pollutants in water.

### 1.1. Dye-Sensitized Semiconductors: From Phototovoltaics to Photocatalysis

Among the variety of semiconductors reported on in the literature, TiO_2_ has been extensively studied in photocatalysis, featuring a great chemical stability, strong redox potential and nontoxicity, together with a high abundance and low cost [10]. However, some key factors prevent this material from being ideal, as it can only be excited with UV radiation due to its wide bandgap (3.2 eV), which limits its photocatalytic efficiency and leads to an insufficient exploitation of sunlight [8]. In addition, TiO_2_-based photocatalysts show a fast charge recombination of the photogenerated excitons (electrons and holes), significantly reducing their overall quantum efficiency. A wide variety of methods has been explored to adequately tune the properties of TiO_2_-based materials. These strategies include the deposition of metal and nonmetal dopants [11] or combination with other semiconductors as cocatalysts to improve charge mobility and modulate the band energy levels for the adjustment of redox capabilities [12]. Control in the fabrication process for the selective production of mesoporous surfaces or defined morphologies can also be used to maximize the surface area, thus, enhancing the catalytic efficiency [13]. Moreover, the sensitization of this semiconductor with organic dyes in order to increase its light absorption has arisen as an exceptional approach, aiming at boosting the photocatalytic capabilities of TiO_2_ [14].

Based on the above, great efforts have been made in the field of dye-sensitized solar cells (DSSCs), where the organic photosensitizer plays a vital role in harvesting solar energy due to a strong visible (and near-infrared) light absorption ability. The benchmark work of Michael Grätzel and Brian O’Regan led the way to the low-cost fabrication of photovoltaic cells with materials of low-to-medium purity, composed of a thin layer of TiO_2_ nanoparticles photosensitized with a monolayer of organic dyes (originally, a polypyridyl complex of ruthenium) [15]. The overall light-to-electric energy conversion efficiency of the as-manufactured DSSCs was reported to be 12% for diffuse daylight, providing large current densities. The long-term stability observed for these DSSCs, together with the possibility of employing printing techniques or surface deposition, drew the attention of manufacturers, making feasible a wide variety of practical applications. Recently, the record of energy conversion efficiency for DSSCs was increased to over 15% under direct sunlight and 30% in scattered light conditions through the cosensitization of two complementary dye molecules [16]. The improvement relies on the fine control in the assembly of the two organic sensitizers on the surface of the titanium dioxide nanoparticle layer, leading to a dense and well-ordered layer of light-harvesting material.

Dye-sensitized photocatalysts (DSPs) were named according to their similarity with DSSCs, both being systems based on the decoration of heterogeneous inorganic photocatalysts with molecular dyes to expand their light-harvesting abilities. Dye sensitizers should be designed in order to promote close contact with the semiconductor (typically TiO_2_), favoring the injection of photogenerated electrons either for electricity generation in DSSCs or for boosting the photocatalytic transformation in DSPs. To allow either the photovoltaic or photocatalytic cycles to continue, the holes (h^+^) generated in the organic dye must be reduced with a suitable hole scavenger to restore the dye to its initial redox state. The role of the so-called sacrificial electron donor (SED) is especially critical in the search for the photoproduction of H_2_ through water splitting [17]. Due to the energetic demands for O_2_ generation and the often rapid back reaction between the photogenerated H_2_ and O_2_, a SED is used for dye recovery, as well as for gaining insights into the intrinsic activity of the photocatalyst.

### 1.2. Organic Dyes as Light-Harvesting Antennas: The Case of Porphyrins

As a result of the research developed in the field of DSSCs, the main organic sensitizers investigated in photocatalysis originate from the experience gained in solar cells, belonging to three main categories: (a) metalorganic complexes based on ruthenium and pyridyl ligands, (b) nonmetallated organic structures and (c) other metal complexes, such as phthalocyanines and porphyrins [18]. Important features to be fulfilled by these organic structures include high light absorptivity and photoconversion efficiency, strong thermal and photochemical stability, a robust and reliable connection with the TiO_2_ surface and adequate oxidation-reduction capabilities. Additionally, a low cost of fabrication and ease of purification are desired for extensive implementations of photocatalytic systems, such as the fabrication of water treatment settings, H_2_ generation plants or large-scale CO_2_ conversion units.

Metalorganic complexes based on d^6^ transition metals such as Ru(II) or Ir(III) complexes with bipyridyl ligands have shown, in general, stable chemical properties, remarkable electron transporting capabilities and prolonged excited states, all ideal features for dye photosensitization [19,20]. However, their laborious and costly synthesis and purification hampers their extensive use. On the contrary, metal-free organic dyes are normally less expensive and, in general, show higher molar extinction coefficients [21]. In this class are xanthene dyes such as rose bengal, rhodamine B, eosin Y and nonmetallic donor–π–acceptor structures able to steer the photoexcited electron donation through the conjugated connection of electron-donating and electron-withdrawing units in the same molecule. Different conjugation patterns such as D–D–π–A and D–A–π–A have been studied, providing improved DSSC efficiencies [22]. However, these organic dyes usually feature a low photoconversion efficiency and a limited chemical stability, which restrain their use as dye sensitizers. Additionally, due to their common aromatic flat structure, xanthene dyes tend to aggregate, affecting the charge carrier transfer ability and impeding the electron donation to the semiconductor.

Porphyrins, naturally present in the photosynthetic apparatus of plants and algae as the core of chlorophyll chromophores, are considered excellent candidates as sensitizers, as they mimic the original photosynthetic function [23]. Due to their aromatic macrocyclic structure with 18 delocalized electrons in the ring, they show a large absorption coefficient in the visible range of light with a Soret band (or B band) centered around 400–450 nm, and a series of Q bands (less intense) positioned between 500 and 700 nm [24]. Structurally related phthalocyanines, constituted by the linkage of four indole units connected by nitrogen atoms, are characterized by their visible/near-IR absorbance ability with intense Q bands between 650 and 800 nm. Based on this light absorption abilities and their remarkable photochemical stability, both porphyrins and phthalocyanines are recognized as outstanding candidates for TiO_2_ sensitizers, featuring great performances as light antennas [24,25]. Notably, they possess an adequate alignment of the lowest unoccupied molecular orbital (LUMO) with the conduction band (CB) of the semiconductor, which allows for an effective electron injection upon photoexcitation. Additionally, porphyrin-based scaffolds constitute a valuable tool in photocatalysis due to the facile modulation of their redox potentials through structural changes in their architecture, providing extensive adaptation capabilities for different photo-driven applications. Other tunable properties related to their structure are light absorption, lifetime of the excited singlet state, stability, aggregation bias and regeneration ability after photooxidation, enabling a complete control of the performance through porphyrin engineering [26]. For all these attributes, these structures are considered powerful candidates for photosensitization, and their performance has recently been proved in the context of H_2_ production [14,18,23,27], photocatalytic degradation of organic pollutants [23] and CO_2_ reduction [28,29,30,31,32,33,34,35,36].

### 1.3. Porphyrin-Sensitized TiO_2_ Photocatalysts: A Matter of Molecular Design

As aforementioned, great progress has been made in the field of DSSCs, where the efficiency of different dye sensitizers has been developed and evaluated considering their operating conditions in combination with iodine-based electrolytes to cyclically regenerate the dye after photooxidation. However, for DSPs, organic dyes must work in very different conditions, such as aqueous environments and, usually, heterogeneous suspensions, which are entirely divergent from the requirements of DSSCs [18]. Another important point is the need for a SED for dye regeneration, which must adequately couple with the HOMO level of the dye. All these factors determine the defined structural and chemical properties for each application and explain why similar organic structures display significantly different performances in DSSCs and DSPs [37]. It is for this reason that further efforts aiming at specifically developing dye sensitizers for DSPs are still needed. In this endeavor, the limitations of porphyrins should be addressed, mainly concerning their stability due to photobleaching, solubility problems (especially in aqueous media), tendency to form aggregates and the subsequent quenching of excited states.

Apart from good light-harvesting properties in the visible region of the electromagnetic spectrum, to ensure that a porphyrin sensitizer performs well, it should smoothly inject the photogenerated electrons into the CB of the semiconductor. A common strategy in this direction consists in effectively achieving the separation of the photogenerated carriers and preventing electron consumption through the undesired recombination of electrons and holes. This can be attained with a D–A structure [38], creating a strong dipole moment and an important push–pull effect, which rapidly drives the migration of electrons toward TiO_2_ upon illumination [39]. At the same time, following this strategy, the injected electron life is extended [40], leading to an increase in photocatalytic activity and a higher quantum efficiency. Along these lines, this review aims at performing an in-depth exploration of the role played by the different architectural motifs of porphyrins in photocatalytic systems stemming from the combination of these photosynthetic pigments with TiO_2_-based nanostructures. While many advances have been achieved in the field of porphyrin-sensitized solar cells [22,41,42,43,44,45,46,47], a structural optimization of these chromophores as sensitizers for DSPs clearly demands a different approach considering the different working conditions in which these dyes must operate. Examining the recent literature, the following analysis intends to find the similarities and the differences between photovoltaic and photocatalytic porphyrin–TiO_2_ hybrid systems to identify the critical factors to take into account in the synthetic design of these pigments. The conclusions drawn from the different sections of this review offer a great opportunity to expand the chemical toolkit in the construction of novel porphyrin-sensitized TiO_2_-based nanostructures, paving the way toward the development of more efficient photocatalysts.

## 2. Porphyrin Architectural Motifs

**Figure 2:**
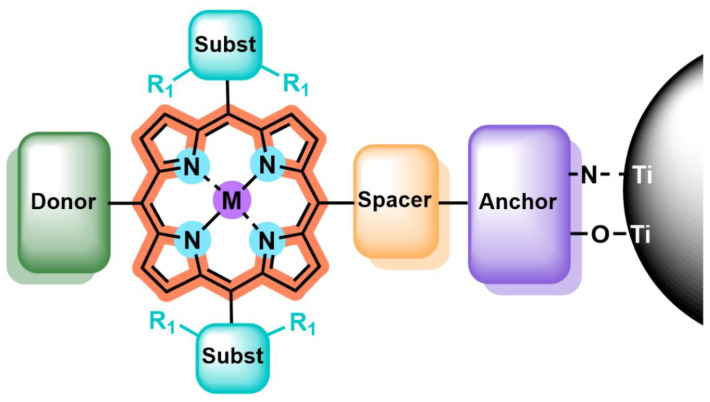
Structural motifs affecting the photocatalytic performance of porphyrin-sensitized TiO_2_ semiconductors: metal center, anchoring group, spacer, peripheral substituents and ring core.

Fabricating efficient TiO_2_ photocatalysts based on the integration of porphyrin sensitizers involves a delicate molecular design, which aims to meet the well-known requirements for dye-sensitized semiconductors, such as a good light-harvesting ability and adequate oxidation and reduction potentials. In this regard, porphyrins constitute highly versatile platforms, as multiple structural motifs can be easily tuned to control the energy bandgap, redox potential, charge injection capability, regeneration efficiency and solubility, among other features. Along the following sections, the main structural modifications and related strategies to enhance the performance of porphyrin sensitizers are overviewed, placing particular focus on their application in the field of photocatalysis. Seeking a better understanding of the issue under discussion, the architectural motifs affecting the physicochemical properties of porphyrins, and, thus, their photosensitizing capabilities, are divided into five different sections: metal center, anchoring group, spacer, peripheral substituents and ring core (Figure 2).

### 2.1. Metal Center

Porphyrins are characterized by the presence of four nitrogen atoms in the core of the aromatic ring, capable of hosting metal ions of a wide variety [48]. The importance of the nature of this metal core is illustrated by the natural porphyrin architectures, which are responsible for performing essential life activities, such as oxygen transport (iron heme and cobalt hemocyanin porphyrins) or photosynthesis and energy transmission in algae and plants (magnesium-chelated chlorophylls) [49]. In all cases, the central metal plays a key role in their activity, constituting, for example, a coordination position for oxygen transport or a metallic core for transferring the photoexcited electrons toward the reaction center.

**Figure 3:**
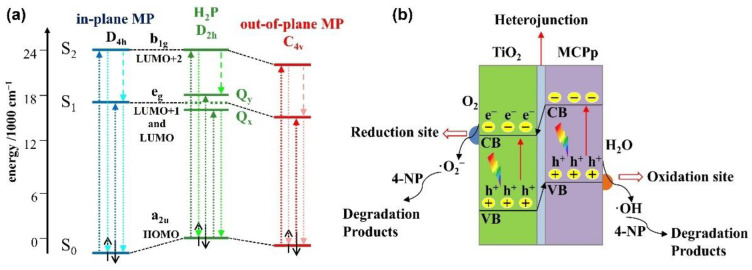
(**a**) Simplified energy level diagram for the change of porphyrin molecular orbitals in different types of complexes. Adapted from reference [51] with permission from Elsevier, copyright 2013; (**b**) photocatalytic mechanism of metalloporphyrin–TiO_2_ hybrid under halogen lamp irradiation. Adapted from reference [52] with permission from Elsevier, copyright 2017.

Depending on the size of the metal cation, different configurations of metalloporphyrins are formed: out-of-plane (or sitting-atop) metalloporphyrins for large cations (with a radius of over 80–90 pm) and in-plane coordinated porphyrins for smaller ions (with a radius of between 55 and 80 pm) [26]. Once bound, the metal center gains electron density from the pyrrole ring through the sigma bonds, and depending on the relative position of the metallic atomic orbitals and the delocalized π-system of the aromatic macrocycle, an electron back transfer takes place. The insertion mode of the metals can be monitored by the changes of the absorption spectra, where redshifts of the Soret band are found for out-of-plane metalloporphyrins (as in the case of Mn^2+^) and blueshifts of the visible absorption bands are characteristic of in-plane metalloporphyrins (as in the case of smaller Fe^3+^ and Cu^2+^ cations) [50]. These phenomena can be explained by the interactions of the atomic orbitals of the central metal with the HOMO and the LUMO of the liganded porphyrin (Figure 3a). When positioned in-plane, overlapping with the HOMO orbital lowers the energy in respect to the HOMO of the metal-free porphyrin, leaving the LUMO level intact. As a result, the HOMO–LUMO bandgap increases and a blueshift is observed. On the other hand, when the metal is positioned out-of-plane, overlapping is maximized with the LUMO orbital, leading to a reduced energy difference with an unmodified HOMO and, therefore, to a redshift of the B band [51]. Notably, the readjustment of the LUMO position and the bandgap between the HOMO and the LUMO of the dye explains why the photoactivity of photosensitized titania with metalloporphyrins is generally higher than the activity shown by free-base porphyrin-sensitized TiO_2_.

Upon metalation, the symmetry of the porphyrin increases, which is reflected in the alteration of the absorption spectra. The resulting coordinated structures present a four-fold rotation axis and, hence, the number of Q bands is reduced to one or two [51,53]. This phenomena was further studied through the calculation of the Mullikan charges of the nitrogen atoms [54], being almost equal for the four nitrogen atoms in the metalloporphyrin due to the covalent interaction between the central metal and the pyrrolic nitrogen atoms. On the other hand, the unequal distribution of charge on the nitrogen atoms of the free-base accounts for the lower symmetry of the porphyrin structure.

The selection of the central ion determines the performance of the metalloporphyrin–TiO_2_ hybrid photocatalyst. In this regard, two key factors should be considered: (1) the number of electrons in the semifilled orbital of the metal and (2) the alignment of the excitation potential, that is, the LUMO energy level, and the CB of TiO_2_ [23]. Along this line, a series of metalloporphyrins was synthesized and tested in the photodegradation of 4-nitrophenol in aqueous media under white light illumination (Figure 3b). Photoreactivity tests showed that the highest efficiency was achieved with the Cu derivative, with the performance order CuP-TiO_2_ > CoP-TiO_2_ ≥ ZnP-TiO_2_ > bare-TiO_2_ [52]. The authors explained the higher efficiency of the Cu(II) catalyst taking into account the d^9^ electronic configuration of the cation, readily gaining an electron to reach the d^10^ steady state. This feature has a great impact in the transfer ability of the photoexcited electrons from the sensitizer to titania, and, correspondingly, in its efficiency as a photocatalyst. Conversely, Zn(II) with a full 3d^10^ configuration reached a relatively stabilized state, and the transfer of an additional photogenerated electron upon the photoexcitation of the porphyrin would become increasingly difficult. The relatively low efficiency of the cochelated sensitizer featuring a d^7^ electronic configuration is probably related to the worse alignment of the LUMO and the CB of TiO_2_.

**Figure 4:**
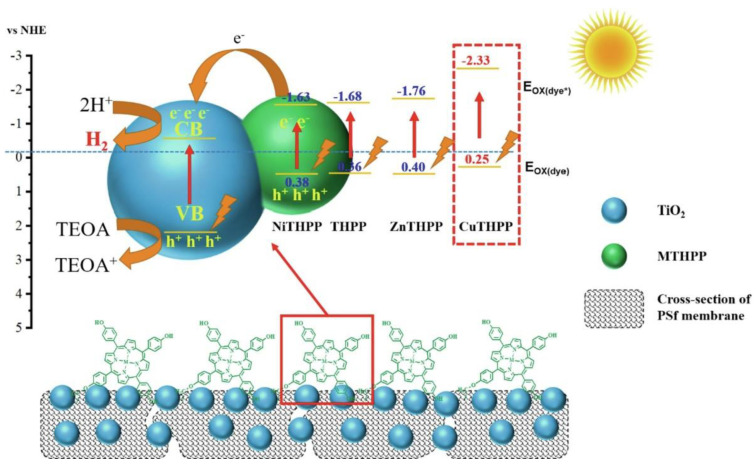
Photocatalytic hydrogen production mechanism: electrons generated by photoexcitation of MTHPP through visible light and TiO_2_ through UV light, enriching the CB of TiO_2_, which promotes the reduction of H_2_O to H_2_ (E_H2/H2O_ = −0.41 eV vs. NHE). Holes (h^+^) on the membrane surface are consumed by TEOA, preventing the recombination of photogenerated e^−^ and h^+^. Reproduced from reference [53] with permission from Elsevier, copyright 2022.

The same trend was recently observed in the use of TiO_2_ membranes sensitized by metal 5,10,15,20-tetra(4-hydroxyphenyl) porphyrins (MTHPP, M = Cu, Zn and Ni) for hydrogen generation induced by UV–visible light (Figure 4) [53]. The metalloporphyrins were covalently anchored on the surface of porous polysulfone (PSf) membranes (M3) loaded with TiO_2_ nanoparticles, avoiding the loss of the photocatalyst during operation. The UV–visible-induced hydrogen evolution from water demonstrated the highest H_2_ production for the Cu metalloporphyrin in the decreasing order CuP-M3 > ZnP-M3 > P-M3 > NiP-M3 using triethanolamine as the sacrificial electron donor. When a full surface coverage of the membrane with the metalloporphyrin was achieved, the hydrogen production reached a maximum of 70.63 mmol/m^2^ after 12 h of illumination. The outperformance of the Cu-based membrane was further studied analyzing the capacity of the photogeneration of electrons under UV–visible light and the excited state oxidation potential, both being key parameters in the electron transfer to the CB of TiO_2_. Based on the cyclic voltammograms and the absorption and emission parameters, the excited state oxidation potentials were calculated to be −2.33, −1.76, −1.63 and −1.68 eV (vs. NHE) for CuTHPP*, ZnTHPP*, NiTHPP* and THPP*, respectively. The Cu metalloporphyrin presented the most negative value, which proved the favorable driving force to rapidly inject electrons in the CB of the semiconductor.

Also applied to hydrogen generation, Huan et al. prepared hybrid materials combining TiO_2_ and porphyrins through a sol–gel method, incorporating Pt nanoparticles [55]. H_2_ production was tested comparing the performance of free-base and metallated mesotetra(4-hydroxyphenyl)porphyrins (Pt/THPP-M-TiO_2_, where M = H, Pd and Zn). The best performance was obtained by the Pd metalloporphyrin (Pt/THPP-Pd-TiO_2_) with the generation of 2025.4 µmol·g^−1^·h^−1^ (12.03 µmol·m^−2^·h^−1^) of H_2_. The higher activity observed was ascribed to the two-center catalyst provided by both the Pt nanoparticles and the central Pd at the porphyrin core. Thus, the Pd porphyrin would act as a dye sensitizer and catalytic active center in the as-prepared hybrid nanocomposites.

The selection of the central metal also determines the preferred self-organization mode, as it was recently demonstrated and exploited for solar hydrogen production [56]. In this work, Pd and Pt tetracarboxyporphyrins (Pd-TCPs and Pt-TCPs) were prepared and adsorbed onto TiO_2_ nanoparticles. Different modes of aggregation were observed, where Pd-TCPs predominantly formed H-aggregates, while Pt-TCPs were organized into J-aggregates. In this work, TiO_2_ played a dual role as the charge transport medium and as the scaffold onto which the porphyrin derivatives self-organize. The authors ascribe the better activity of Pt-TCP for hydrogen production to the self-organization into J-aggregates, which are known to favor electron transport as they induce stronger electronic coupling between porphyrins. Thus, upon excitation, a first electron transfer occurs, and a platinum hydride species is formed (Pt(III)-H). Next, the formed aggregates transfer electrons to the CB of TiO_2_, followed by a second electron transfer to the active site: the platinum hydride. Finally, the uptake of a proton leads to the formation of molecular hydrogen and the restoration of Pt(II).

Another important issue, especially in reactions in the gas phase, is the fixation of the molecules to be transformed at the active site. A paradigmatic example is the reduction of CO_2_. In this sense, transition metal porphyrins show a remarkable ability to capture CO_2_ through direct coordination with the metallic core [49]. Additionally, the central location in the aromatic porphyrin ring is ideal for catalytic activity, promoting the electron transfer from the backbone of the porphyrin to the CO_2_ molecule through the metal center. This was recently demonstrated by Ma et al., who described the construction of a composite photocatalyst endowed with a porphyrin-based polymer built onto a hollow TiO_2_ surface [32]. The coordination of Pt(II) with the pyrrolic nitrogen’s demonstrated to be determinant in the selective CO_2_/O_2_ adsorption and, hence, in the photocatalytic reduction of CO_2_ under ambient conditions. The mechanism proposed for the photocatalytic activity of this composite was based on the photogeneration of electrons into the conduction band of TiO_2_ upon irradiation with a 300 W xenon lamp and the rapid transfer to the Pd(II) metalloporphyrin center, where the adsorbed CO_2_ is located. Thus, the reduction to CH_4_ and CO occurs while the holes in the valence band of TiO_2_ are quenched through the oxidation of adsorbed water in the hollow TiO_2_. In this system, the central Pd(II) plays a dual role, selectively fixing CO_2_ versus O_2_ and efficiently promoting the reduction reaction, contributing to charge separation and the final electron transfer.

**Figure 5:**
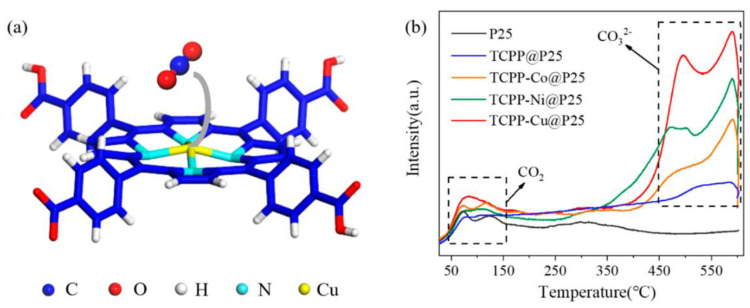
(**a**) Optimized structure for CO_2_ adsorption on a TCPP-Cu unit; (**b**) CO_2_-TPD spectra of P25, TCPP@P25, TCPP-Co@P25, TCPP-Ni@P25 and TCPP-Cu@P25. Reproduced from reference [57] with permission from MDPI, copyright 2020.

Additionally, Wang et al. explored the use of alternative metalloporphyrins for CO_2_ reduction, incorporating either Co, Ni or Cu in the center of 5,10,15,20-tetrakis(4-methoxycarbonylphenyl)porphyrin (H_2_TCPP) (Figure 5a) [57]. The as-obtained metalloporphyrins were loaded onto commercial titania (P25) to form a TCPP-M@P25 (M = Co, Ni and Cu) catalyst. The resulting nanocomposites showed a remarkably enhanced photocatalytic activity in comparison with the free-base porphyrin@P25 catalyst. This enhancement was attributed both to the promoted electron transfer from the photoexcited semiconductor to the metal center of the porphyrin ring and the affinity of CO_2_ molecules to the metallic core. Interestingly, the authors studied the adsorption of CO_2_ using temperature-programmed desorption (TPD) (Figure 5b). The results obtained indicated that the presence of a coordinated metal in the porphyrin ring correlated with a significant increase in the intensity of desorption peaks at 380–550 °C and 550–600 °C, which was ascribed to the desorption of bidentate carbonates (b-CO_3_^2−^) and monodentate carbonates (m-CO_3_^2−^), respectively. These findings suggested that the central metal remarkably promotes the chemisorption of CO_2_, being the Cu-based catalyst showing higher intensities. These data are in line with the highest evolution rates of CO (13.6 µmol·g^−1^·h^−1^) and CH_4_ (1.0 µmol·g^−1^·h^−1^) obtained with the TCPP-Cu@P25, which were 35.8 times and 97.0 times those of bare P25, respectively.

**Figure 6:**
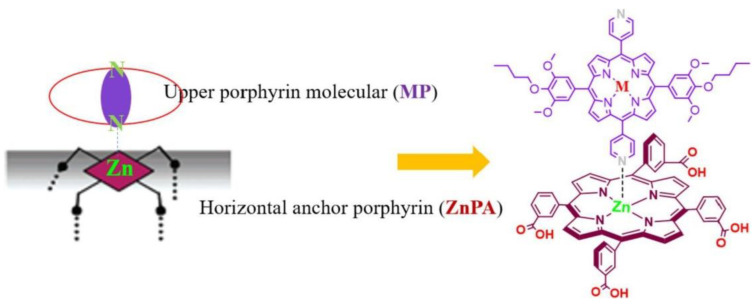
Detailed building approach of the assembly of a metalloporphyrin (MP) and an anchoring Zn porphyrin (ZnPA) on a TiO_2_ electrode surface. Reproduced from reference [58] with permission from Elsevier, copyright 2022.

Different molecular architectures have been explored in the field of DSSCs for improving light-harvesting abilities. Taking advantage of the axial coordination capacity of the central Zn ion, Wu et al. designed novel self-assemblies of porphyrins, where a horizontal Zn porphyrin (ZnPA) was used as the first anchor layer to which a pyridine-functionalized metalloporphyrin (MP where M = Zn, Cd, Ag and Cu) coordinated to form an upper layer of porphyrins (Figure 6) [58]. This metal–ligand axial coordinating approach proved to be superior over the single horizontal porphyrin, attaining an enhanced light-harvesting ability and higher current densities (*J_SC_*) with the double-layer architecture. The incident photon-to-current conversion efficiency (IPCE) spectra exhibited very similar results for all the MP-ZnPA dyes tested. However, increasing values were observed following the order ZnPA < CuP-ZnPA < CdP-ZnPA < ZnP-ZnPA < AgP-ZnPA. Additionally, the photocurrent density–voltage curves (J–V) and photovoltaic parameter analysis pointed out the better performance of AgP-ZnPA. In contrast, the electronically analogous CuP-ZnPA sensitizer showed lower current intensities, probably due to the high exciton recombination rates.

**Figure 7:**
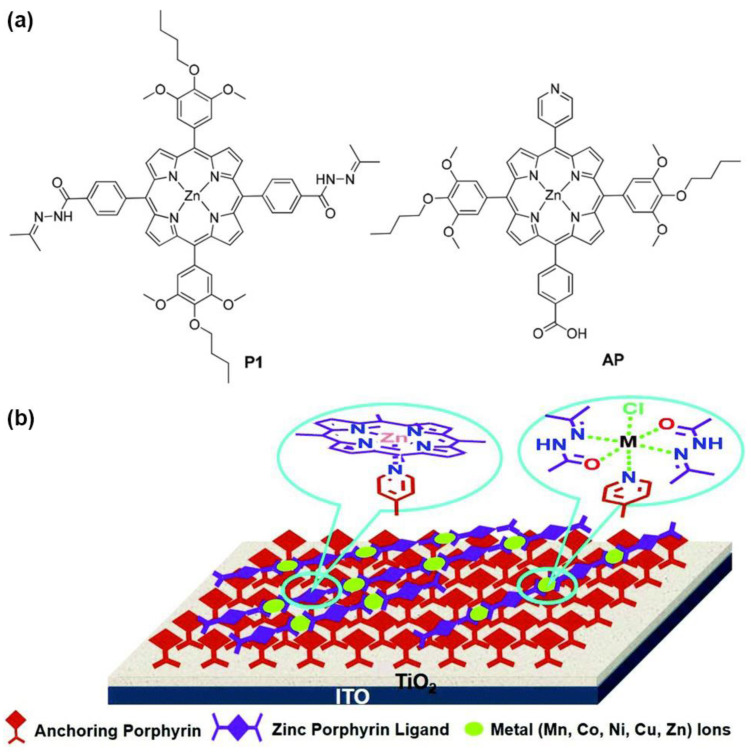
(**a**) Chemical structures of porphyrin ligand P1 and anchoring porphyrin AP; (**b**) modes of axial coordination-bond-assisted bilayer chromophores on TiO_2_ electrode surfaces. Adapted from reference [59] with permission from the Royal Society of Chemistry, copyright 2016.

Another example exploiting the axial coordination ability of Zn metalloporphyrins for enhanced light harvesting in DSSCs was described by Zhang et al. [59]. Their proposed design consisted of a bilayer supramolecular structure with a first layer of anchoring porphyrins (APs) endowed with a pyridine substituent capable of coordinating to a second layer of Zn-metallated porphyrins (P1) linked to each other through a Schiff base structure (Figure 7). With this design in mind, the authors synthesized Zn porphyrins featuring an acylhydrazone as the linking group to build up the coordination polymers (CPs) with different metal ions, namely, Mn^2+^, Co^2+^, Ni^2+^ and Zn^2+^. The advantage of this architecture lies in the facile construction of supramolecular donor–acceptor systems through coordination interactions, avoiding the complicated and high-cost synthesis of porphyrins. The incorporation of donor substituents in the coordination polymer and the acceptor groups in the anchoring porphyrin would lead to a D–A structure, promoting charge separation and an effective electron transfer from the porphyrin donor to the TiO_2_ surface through the axial ligand. Although the cell performance of the bilayer structure suffered from charge recombination problems at the dye/TiO_2_/electrolyte interfaces, the systematical coordination approach developed in this work illustrates the possibility of easily constructing novel self-assembled supramolecular structures for light harvesting.

**Figure 8:**
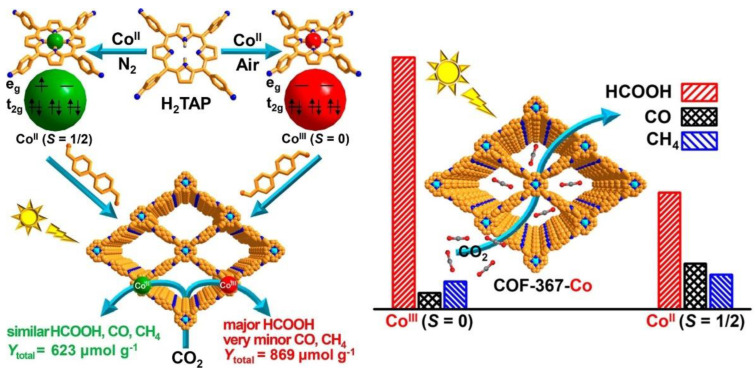
Rational fabrication of COF-367-Co featuring different spin states of Co ions toward photocatalytic CO_2_ reduction. Adapted from reference [60] with permission from the American Chemical Society, copyright 2020.

Also related to the nature of central metals, spin states and the distribution of electrons in d orbitals have had an influence on the evolution of intermediates in catalyzed processes. As a consequence, the activity and selectivity of a photocatalyzed reaction can be modulated. Along these lines, Gong et al. recently reported on the preparation of COFs (COF-367-Co) featuring Co centered in porphyrin units (Figure 8) [60]. The spin state of cobalt was manipulated by regulating its oxidation state. Thus, Co(II) species were incorporated in the porphyrin centers and the initial Co(II) was oxidized to Co(III) in the air, keeping the overall structure intact. Photocatalytic CO_2_ reduction experiments showed that COF-367-Co(III) (spin = 0) demonstrated higher activity and exceptionally higher selectivity, producing almost exclusively HCOOH in comparison to the performance shown by COF-367-Co(II) (spin = 1/2).

In order to disclose the key factors that modulate the different reactivity of Co(III) and Co(II), DFT calculations were performed revealing a stronger binding with CO_2_ molecules by the low spin Co(III). The different electron distribution of the Co-3d orbitals (spin state) in both species directly affected the binding mode. Thus, Co(III) coupled with O-2p via an overlapping interaction with Co-3d_z_^2^ in a very effective manner, while Co(II) used Co-2d_xz_ or Co-3d_yz_, which was less efficient. Additionally, DFT calculations described a lower energy barrier for HCOOH formation and a higher energy barrier for HCOOH in the case of the COF-367-Co(III)-photocatalyzed process, in comparison with COF-367-Co(II). These results supported the experimental selectivity observed, where the subsequent conversion of HCOOH to generate CO and CH_4_ in COF-367-Co(III) was disfavored.

In conclusion, the selection of a metal center in metalloporphyrins can have a remarkable impact on both the optoelectronic properties and the photocatalytic performance of the porphyrin–TiO_2_-based composites. The electronic distribution in the central metallic ion determines the interaction with the porphyrin ligand and the reaction substrate, which have an enormous influence on the activity and selectivity of the photocatalyzed process. Hence, the size of the metal ion, valence, spin and oxidation states may cause important differences, both in the photoredox features and in the coordination capabilities. All these factors can determine the evolution of reaction intermediates and the efficiency of the photocatalyzed transformation.

### 2.2. Anchoring Group

A determining factor in the photocatalytic performance of DSPs stems from the interface connection between the dye and the TiO_2_ material. Taking advantage of the abundant hydroxyl groups of the nanosized TiO_2_ [61], different kinds of anchoring groups have been developed. These linkers play a dual role, ideally providing a robust dye–semiconductor connection and favoring electron injection under light excitation. Great efforts have been contributed in order to optimize the immobilization of dye sensitizers onto metal oxide substrates in the context of DSSCs [62]. Although the attachment is usually performed with covalent bonds, thus, affording a strong coupling and device stability, other types of interactions have been explored, including hydrogen bonding, electrostatic interactions, hydrophobic interactions and van der Waals forces.

**Figure 9:**
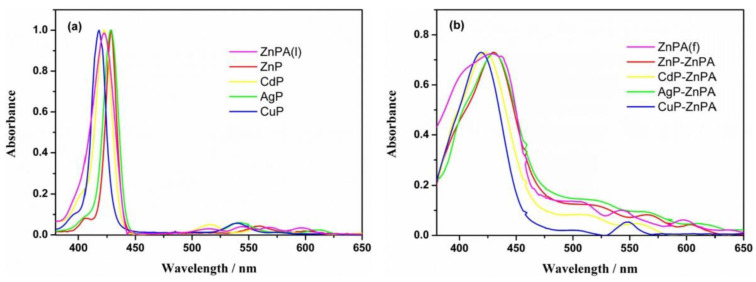
UV–visible absorption spectra of different metalloporphyrins (**a**) in CH_2_Cl_2_ solution and (**b**) on TiO_2_ thin films. Reproduced from reference [58] with permission from Elsevier, copyright 2022.

Adsorbed porphyrins on TiO_2_ typically show redshifted and broader B and Q absorption bands upon binding to the semiconductor surface [63,64,65], which is indicative of the interaction between the porphyrin ring and the polar surface of the semiconductor (Figure 9). Further information on the molecular structure of the bound porphyrins can be obtained with Fourier-transform–infrared spectroscopy (FTIR), comparing the changes of the vibrational bands before and after the interaction with the TiO_2_ surface [24,63,66,67,68]. Thus, C=O stretching vibrations at 1629 cm^−1^ of *meso*-tetrakis(4-carboxyphenyl)porphyrin (TCPP) shifted to higher wavenumbers (1642 cm^−1^) upon binding to the TiO_2_ colloidal surface [63]. Similarly, when sulfonic acid and methoxy groups were selected as the anchoring functionalities, alterations in the characteristic vibrational bands of these functional groups were observed, indicating that the interaction between the porphyrins and TiO_2_ surface indeed occurred through these anchoring groups.

For the covalent attachment approach, carboxylic and cyanoacrylic acid anchor groups are traditionally employed. In this case, apart from providing the necessary union with the semiconductor surface, they also play a role as accepting groups. This is especially important in D–π–A-type structures, where the molecular dipole moment orientates the electron transfer from the donor part of the sensitizer toward the TiO_2_ surface through the electron-withdrawing anchoring group. Additionally, the strong polarization of the organic sensitizer decreases the back electron transfer from the semiconductor to the molecular dye. This feature explains the great cell performance observed for dyes attached through carboxyl moieties in comparison to other functional groups.

**Figure 10:**
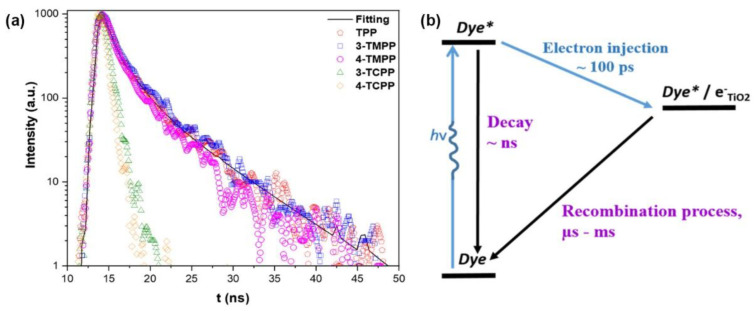
(**a**) Time-resolved emission decay of free-base porphyrins fitted with lifetime distribution fitting function and (**b**) diagram of the electron transfer mechanism, showing the competitive electron transfer that occurs in the DSSC photoanode. TPP = tetraphenylporphyrin; 3-TMPP = (3-tetra(methylphenyl)porphyrin; 4-TMPP = (4-tetra(methylphenyl)porphyrin; 3-TCPP = (3-tetra(carboxylphenyl)porphyrin; 4-TCPP = (4-tetra(carboxylphenyl)porphyrin. Adapted from reference [65] with permission from Wiley, copyright 2020.

The stronger electron coupling between porphyrins and TiO_2_ surfaces promotes the electron injection from porphyrins, which can be observed through time-resolved emission spectroscopy. These measurements provide information on the electron transfer dynamics, measuring the electron injection rate from the porphyrin excited state to the CB of TiO_2_. In this regard, Milana et al. studied the differences in electron transfer rates depending on the type of interaction with the titania surface [65]. Figure 10 shows a remarkable faster emission decay for porphyrins featuring a carboxylic group as the anchoring unit (3-TCPP and 4-TCPP) in comparison to the weakly physisorbed tetraphenylporphyrin (TPP) and methylated derivatives (3-TMPP and 4-TMPP). These results confirm the stronger connection to the TiO_2_ surface through the carboxylic acid and the subsequent enhancement in the electronic injection rate. However, the cyanoacrylic acid and carboxyl groups present relatively poor long-term stability, being prone to dissociate under aqueous and alkaline conditions through the hydrolysis reaction of the ester linkage between the dye and the TiO_2_ surface [69]. This process results in the loss of cell performance and important problems concerning device durability. In terms of photocatalytic applications, this is a particularly determining factor to consider in aqueous operating devices such as water-splitting systems or water-purifying units. Moreover, binding moieties that can undergo *trans*-to-*cis* photoisomerization, a process that competes with electron injection, result in the reduced efficiency of these devices [70]. As a consequence of these issues, alternative linkers have been explored [69,71]. In this context, phosphonic acids have been described as robust anchoring groups with a much stronger affinity to TiO_2_ [62]. The binding modes of this anchoring group make use of both Lewis acidic sites on the TiO_2_ surface and condensation reactions with superficial hydroxyl groups. This functional group provides great stability, with desorption not detected in the aqueous solution. However, the charge transfer ability of this group was much lower than that observed for carboxylic acids, which was due to the tetrahedron geometry of the central phosphorous atom and the loss of conjugation [72]. Siloxyl connections were also explored as robust anchoring groups [69], and silatrane functionalities were introduced as convenient triethanolamine-protected trialkoxysilanes, which readily deprotected via the nucleophilic attack of the surface hydroxyl groups forming the desired siloxyl bonds. The bi- or tridentate linkages formed with the TiO_2_ surfaces were significantly less labile than the ester bonds, providing a durable connection between the dye and the semiconductor even under basic hydrolysis conditions.

**Figure 11:**
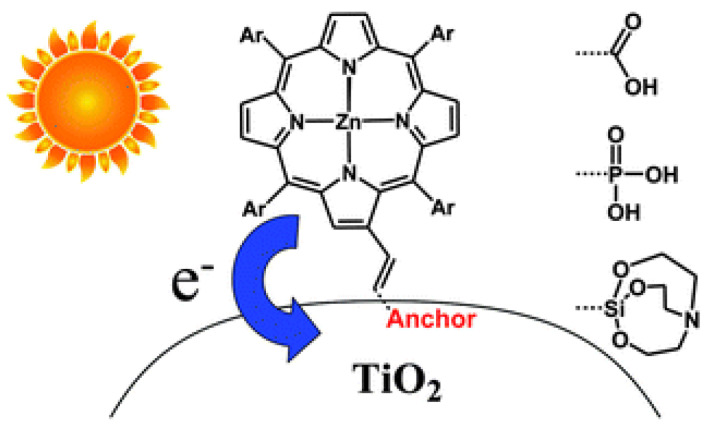
Structure of tetra-arylporphyrin dyes functionalized with three different anchoring groups (carboxylic acid, phosphonic acid and silatrane) for TiO_2_ binding. Reproduced from reference [69] with permission from the Royal Society of Chemistry, copyright 2013.

**Figure 12:**
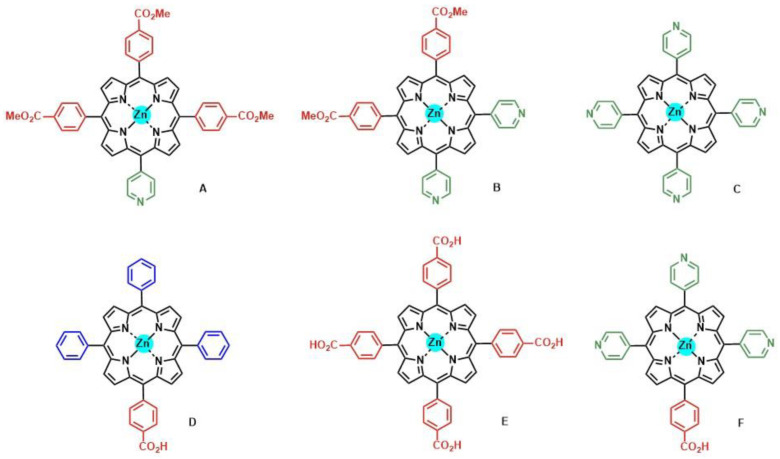
Molecular structures of the series of Zn porphyrin dyes reported by Daphnomili et al. (**A**–**C**) [66] and Calmeiro et al. (**D**–**F**) [64].

The photoelectrochemical performance was compared between silatrane-, phosphonic-acid- and carboxylic-acid-derived porphyrin sensitizers in DSSCs (Figure 11) [69]. The authors found that the surface coverage of the carboxylic acid sensitizer was approximately double the functionalization achieved with either the phosphonic acid or the silatrane porphyrins, which was ascribed to the different surface requirements derived from the sp^3^ character of the central atom in phosphonate and siloxyl linkages. The solar conversion efficiency of the carboxylic acid sensitizer was twice higher in comparison with those observed with the phosphonic acid and silatrane linkers, which was probably due to the increased surface coverage. Therefore, a comparable photoelectrochemical performance was obtained with more stable anchoring groups such as phosphonate and siloxyl functionalities. Pyridyl anchoring groups were also tested for the attachment of porphyrins onto TiO_2_ surfaces in the context of DSSCs [67,68,73]. In this case, the bonding between the pyridine ring and the semiconductor took place on the Lewis acid sites of the TiO_2_ surface, leading to an efficient electron injection. Daphnomili et al. tested the performance of a series of Zn-porphyrin dyes, featuring one, two, and four pyridine rings at different *meso*-positions (Figure 12A–C) [66]. Porphyrins with two pyridyl substituents in *cis* relative positions (B) demonstrated better photoelectrochemical properties compared to those found for the other pyridine-functionalized porphyrins of the series. The difference was attributed to a higher dye loading and a more adequate arrangement of the HOMO and LUMO energy levels, giving rise to an efficient electron injection and dye regeneration. An assessment of the binding capacity of pyridyl in comparison to carboxyphenyl acid moieties was illustrated in the results recently reported by Calmeiro et al. (Figure 12C–F) [64]. These authors described a very low intensity of the absorption spectra of tetrapyridyl porphyrin C loaded onto TiO_2_ films. These dyes lacked carboxylic groups for anchorage and exclusively relied on the coordination bonds between the pyridyl nitrogen atoms and the Lewis acidic sites on the TiO_2_ surface.

**Figure 13:**
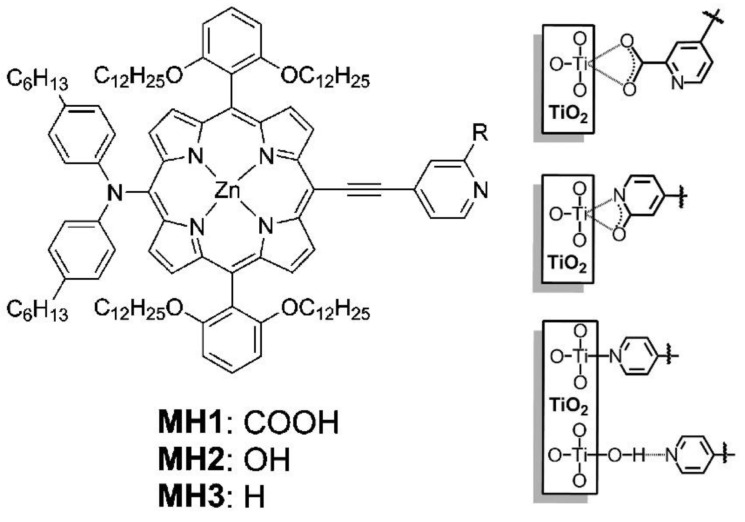
Molecular structures of Zn porphyrins designed by Mai et al. and binding modes of 2-carboxypyridine, 2-pyridone and pyridine anchoring groups on the TiO_2_ surface. Adapted from reference [68] with permission from the American Chemical Society, copyright 2015.

Similar results were obtained by Kumar et al. [73], who studied a series of Zn-thienyl porphyrins decorated with different anchoring groups, including pyridyl, hydroxyphenyl and carboxyphenyl functionalities. The dye loading of the pyridyl- and hydroxyphenyl-connected porphyrins was lower than the analogous structure featuring a carboxyphenyl anchoring group. However, the incorporation of an additional functional group reinforcing the binding ability of the pyridine-based anchoring groups resulted in a robust linkage of the dye to the TiO_2_ surface. This strategy was explored by Mai et al. [68], demonstrating that 2-carboxypyridine could provide a stable bond between the dye and TiO_2_, leading to an even better cell performance and long-term stability than that observed for the analogous dye displaying a 4-carboxyphenyl anchoring group (YD2-o-C8) (Figure 13).

**Figure 14:**
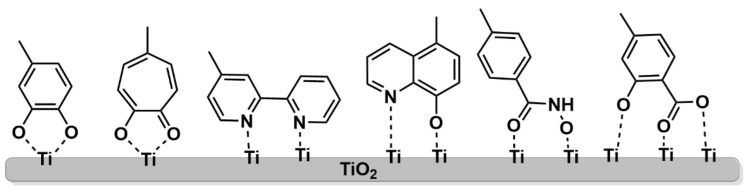
Postulated modes of binding of catechol [74], tropolone [76], bipyridyl [67], 8-hydroxyquinoline [75], hydroxamic acid [77] and salicylic acid [78] anchoring groups on TiO_2_ surfaces.

Other robust anchoring groups based on a bidentate coordination pattern have been developed (Figure 14), such as catechol [74], 8-hydroxyquinoline [75], bipyridyl [67], tropolone [76], hydroxamic acid [77] or salicylic acid [78], the latter endowed with a tridentate binding ability, showing in all cases an enhanced DSSC durability and binding capacity.

The orientation of the π-macrocycle of the porphyrin ring with respect to the TiO_2_ surface also has a strong impact in the electron transfer process between the dye and the semiconductor. A flat orientation of the porphyrin brings the π-system closer to the TiO_2_ surface, accelerating the electron transfer from the excited dye to the CB of the semiconductor.

**Figure 15:**
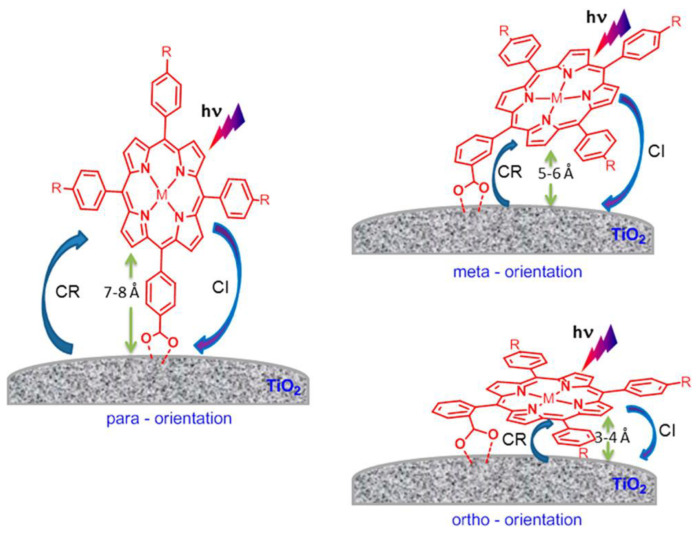
Relative orientation of *para*-, *meta*- and *ortho*-carboxyphenyl functionalized porphyrin adsorbed on a TiO_2_ surface. Key photochemical events responsible for cell performance are also shown (CI, charge injection; CR, charge recombination). Reproduced from reference [79] with permission from the American Chemical Society, copyright 2013.

The effect of macrocycle orientation on the DSSC performance was studied by Hart et al. by comparing zinc porphyrins bearing a carboxyl-anchoring group at the *para*-, *meta*- and *ortho*-positions of the phenyl ring at the *meso*-positions (Figure 15) [79]. Due to the bidentate nature of the binding of the carboxyl moiety, the *para*-derivatives predominantly placed the aromatic macrocycle orthogonal to the TiO_2_ surface. However, the *meta*- and *ortho*-substituted phenyl rings provided a more tilted arrangement, positioning the π-system at an angle between 50° and 80° for the *meta*-porphyrin derivative and an almost flat orientation to the TiO_2_ surface for the *ortho*-substituted porphyrin. In terms of cell performance, the *para*- and *meta*-derivatives showed better results, probably due to the fast charge recombination in the case of *ortho*-derivatives, seemingly via a through-space electron transfer mechanism.

**Figure 16:**
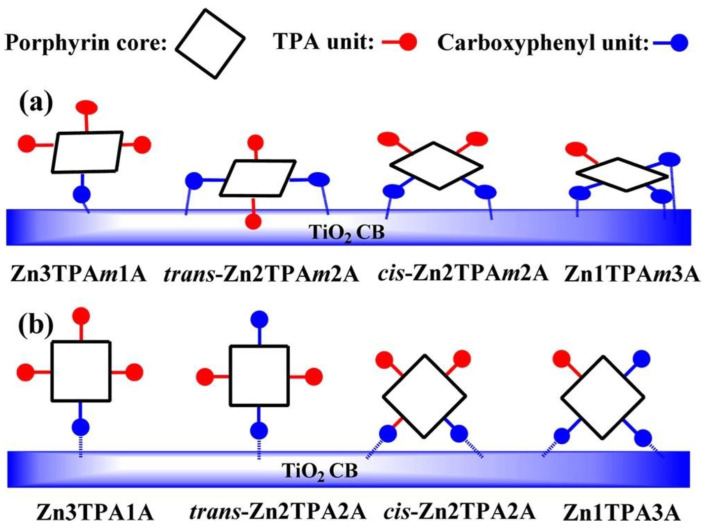
Possible modes of attachments of (**a**) *meta*- and (**b**) *para*-series of porphyrins on TiO_2_ surface. Members of each series featured increasing numbers of carboxyl-anchoring units (depicted from left to right). TPA = triphenylamine unit. Reproduced from reference [80] with permission from the American Chemical Society, copyright 2015.

Additionally, the orientation of the porphyrin rings could be controlled with the number of substituents [39,80,81]. In this line, Ambre et al. evaluated the number of carboxyl units attached to TiO_2_ in a series of Zn porphyrins with an increasing number of *meta*- or *para*-carboxyphenyl anchoring groups [80]. By means of the analysis of the ATR-FTIR spectroscopy differences of the adsorbed porphyrins in comparison to those found for neat powders, these authors deduced different modes of attachments of *meta*- and *para*-carboxyphenyl series of porphyrins on the TiO_2_ surface (Figure 16). In the *meta*-series, all porphyrins attached to the TiO_2_ oriented on the surface with tilt angles larger for the mono- and *cis*-functionalized porphyrins, whereas the *trans*- and tri-substituted dyes laid flat on the TiO_2_ surface. The *para*-series showed in all cases an orthogonal orientation in respect to the surface, attaching to the semiconductor surface either by one or two carboxyl anchors depending on the number and relative positions of the substituents. The tilted and flat orientations of the *meta*-derivatives placed the central zinc close to the TiO_2_ surface, favoring the recombination of the electrons in the CB of TiO_2_ with the oxidized porphyrins. Notably, the *para*-series showed an enhanced DSSC performance due to the perpendicular disposition, which allowed for an effective electron transfer over a detrimental charge recombination.

Keawin et al. prepared three porphyrins as sensitizers in DSSCs showing either one, two or three carboxylic acid groups linked to the porphyrin core through a phenyl–ethynyl–phenyl spacer [81]. The results obtained in this study pointed to a decreasing conversion efficiency as the numbers of anchoring groups on the porphyrin ring increased. DFT calculations indicated that the electron-withdrawing effect due to the presence of additional (two or three) carboxyl moieties had an electronic effect on the π-macrocycle, hindering the electron injection into the TiO_2_ conduction band.

**Figure 17:**
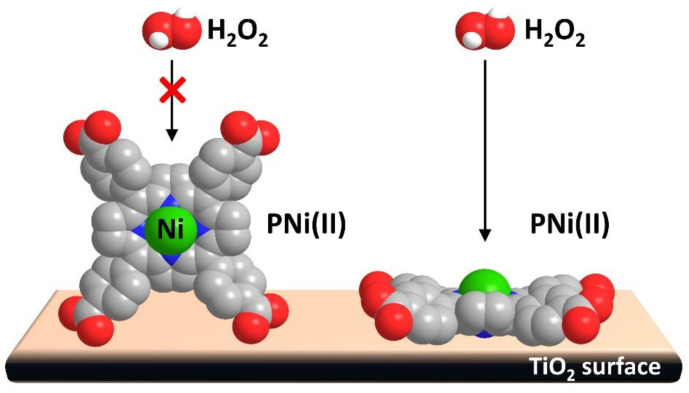
Nickel tetra(4-carboxyphenyl)porphyrin (TCPPNi) geometry and interaction with TiO_2_ surface and H_2_O_2_. Adapted from reference [82] with permission from Elsevier, copyright 2016.

The effect of the anchoring mode on the activity was also studied in the context of photocatalysis [82,83]. Tasseroul et al. demonstrated the dependence of the anchoring behavior on TiO_2_ with the concentration of nickel tetra(4-carboxyphenyl)porphyrin (TCPPNi) [82]. At low TCPPNi concentrations, the anchoring proceeded through the four carboxylic groups, while at higher concentrations, the attachment took place by only one or two carboxylic anchors, adopting an edge stacking disposition (Figure 17).

In order to evaluate the activity of the catalyst depending on the amount of porphyrin adsorbed, the degradation of hydrogen peroxide was used as a test reaction. The authors found a direct relationship between the catalytic activity and the anchoring mode. Thus, at low concentrations (optimal 0.0115 mol of porphyrin per gram of Degussa P25 TiO_2_), the flat geometry of the dye resulted in the promotion of H_2_O_2_ degradation. In this disposition, the central Ni atom seemed to be more accessible to the reactant, and the low amount of sensitizer covered less than 50% of the TiO_2_ surface. Therefore, the direct UV photoactivation of the semiconductor could also contribute to the performance of the catalyst.

**Figure 18:**
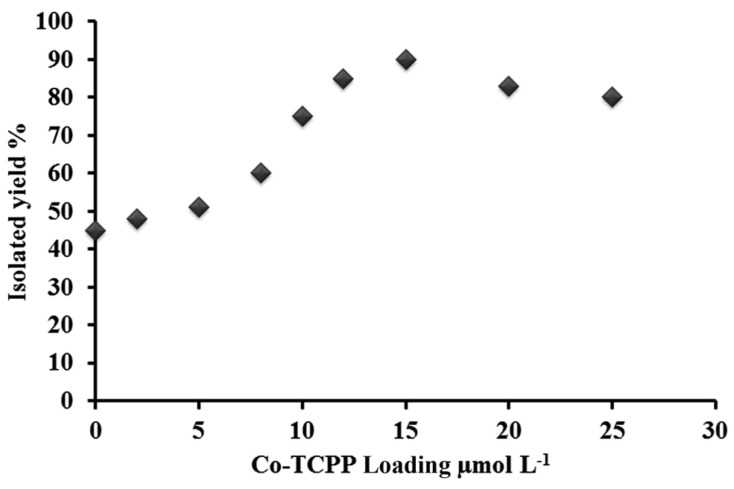
Effect of Co-TCPP loading on the activity of the oxidation reaction of benzaldehyde. Reproduced from reference [83] with permission from the Royal Society of Chemistry, copyright 2016.

Safaei and Mohebbi explored the photocatalytic behavior of a TiO_2_/WO_3_ nanocomposite sensitized with cobalt(II)-*meso*-tetra(4-carboxyphenyl)porphyrin (Co-TCPP@TiO_2_/WO_3_) for the selective oxidation of primary alcohols under visible light irradiation, using air as the oxygen source [83]. In this case, a dependence of the catalytic performance on the loading of the Co porphyrin was also observed (Figure 18), showing an increased conversion of benzyl alcohol with the increase in porphyrin loading up to 11.7·10^2^ μmol·L^−1^. At higher loadings, the yield of oxidized benzaldehyde decreased, which was ascribed to the formation of H-aggregates. These aggregates presented a lower electron transfer efficiency for the charge transfer to TiO_2_ CB, probably due to the competitive nonradiative deactivation of the excited porphyrins.

Alternative anchoring modes have been explored, taking advantage of the known ability of pyridine to establish axial coordination with zinc porphyrins. Martini et al. studied the relative injection efficiencies of surface-bound pyridine linked to TiO_2_ via four different moieties: carboxylate, phosphonate, acetylacetonate and hydroxamate [71]. Following this approach, both the optimization of the electronic properties of the dye porphyrin and the anchoring group could be performed independently. To assess the potential use of this anchoring strategy in water-splitting technologies, the stability of these anchoring pyridines was studied in aqueous conditions. The results showed that the hydroxamate linkers were stable in water for prolonged times, and their electron injection efficiency was comparable to the carboxylate linkers’ performance. Likewise, the authors compared the relative efficiency of the electron injection of porphyrins bound through the pyridine linker with those directly bonded via the carboxylate bidentate connection. A comparison of data collected based on time-resolved THz spectroscopy (TRTS) indicated similar electron injection efficiencies of the photoexcited sensitizers into the conduction band of the TiO_2_ nanoparticles.

**Figure 19:**
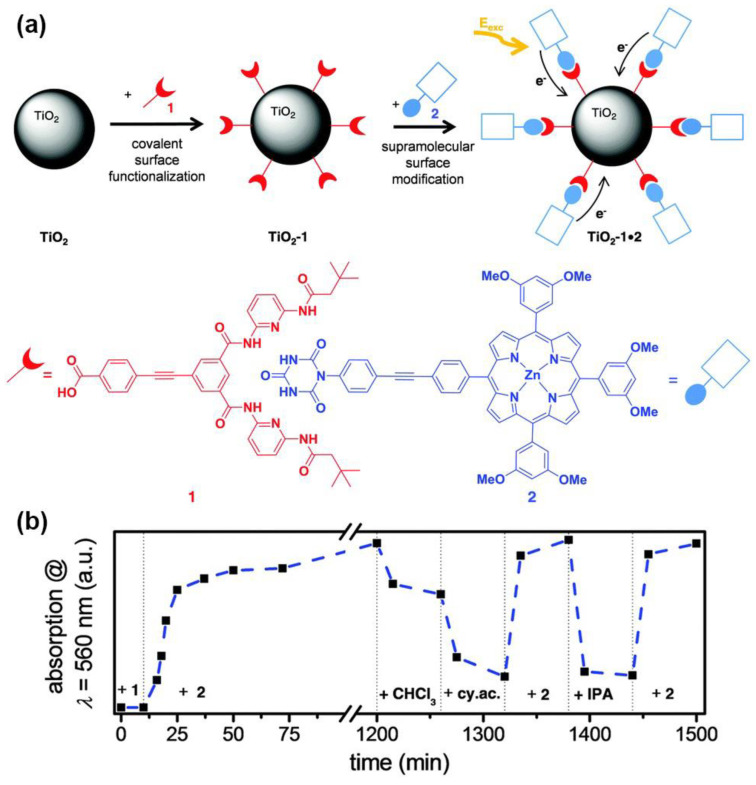
(**a**) Conceptional sketch of the supramolecular self-assembly of porphyrins onto TiO_2_ nanoparticles by means of hydrogen bonding interactions and (**b**) time-dependent UV–visible study for an illustration of the reversibility of the self-assembly of porphyrin 2 on Hamilton receptor-modified TiO_2_ layers (absorption at 560 nm vs. time). Adapted from reference [84] with permission from the Royal Society of Chemistry, copyright 2016.

A supramolecular anchoring mode was described using the hydrogen bonding Hamilton receptor, featuring six-point hydrogen bonding interactions [84]. This motif easily binds cyanurates with association constants from 10^3^ to 10^6^ M^−1^ in apolar media. In this work, Zeininger et al. reported on the successful noncovalent grafting of porphyrin sensitizers onto the surface of TiO_2_ nanoparticles (Figure 19a). The reversibility of this anchoring mode was also demonstrated through time-dependent absorption studies (Figure 19b). The loading of porphyrin was achieved by immersing TiO_2_-coated glass slides modified with the Hamilton receptor in porphyrin chloroform solutions, while desorption was efficiently performed using isopropanol or a solution of competing cyanurate instead.

Although DSSCs constructed with this alternative mode of attachment showed a rather low efficiency, this work provides the chemical tools necessary to reversibly adsorb chromophores onto a semiconductor surface, gaining control in the replacement of sensitizers in operating devices and further functionalizing opportunities.

In summary, the anchoring group constitutes a key element in the design of sensitizers for photocatalytic transformations. This group should provide a robust connection to the semiconductor, always paying special attention to the reaction conditions required for the photocatalyzed process. At the same time, this moiety should strongly favor the electron injection from the photoexcited sensitizer to the conduction band of TiO_2_. Although carboxylic acids have been traditionally used, other functionalities offer enhanced durability and binding abilities, such as the bidentate coordination of 8-hydroxyquinoline, catechol, hydroxamic acid or tridentate salicylic acid. In addition, a delicate control of the orientation of the sensitizer in respect to the semiconductor surface can become crucial, especially in those cases where reactants need to reach the porphyrin core in order to evolve. This can be controlled by the number and position of anchoring functionalities, as described above.

### 2.3. Spacer

Over recent years, the high cost and low availability of Ru(II) polypyridyl complexes, together with their lack of absorption in the near IR region, have urged the search for cost-effective alternatives that can act as panchromatic sensitizers. Following this principle, Grätzel’s group reported in 2016 their results on the redesign of new *meso*-substituted D–π–A porphyrin structures incorporating a benzothiadiazole (BDT) conjugate spacer [85]. The resulting dye featured a significant broadening of the Soret and Q bands, providing better optical performance both in the green and red regions of the spectrum. DSSCs based on this sensitizer provided an unprecedent 13% PCE, without the addition of a cosensitizer.

The introduction of the BDT unit had a remarkable impact on the absorption spectrum of porphyrin dyes, leading to a split in the Soret band and a redshift in the lowest-energy Q band. Additionally, the enhanced molar absorptivity of the redshifted Q band was ascribed to the increased conjugation and polarizability along the donor–acceptor axis resulting from the incorporation of the π-conjugated spacer. Notably, with the introduction of this BDT auxiliary acceptor between the porphyrin ring and the acceptor-anchoring group, the absorption gap between the Soret band and the Q band could be efficiently filled, avoiding the need for complementary cosensitization.

**Figure 20:**
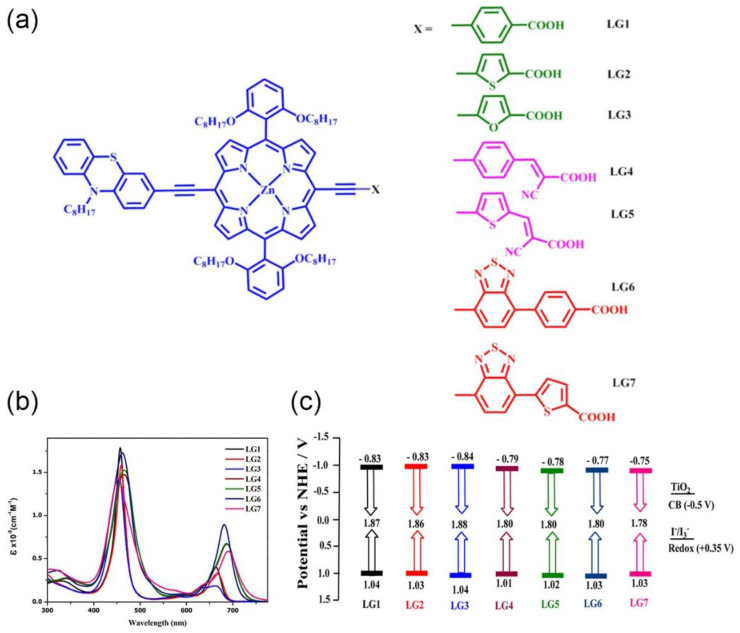
(**a**) Molecular structures of porphyrin sensitizers; (**b**) UV–visible absorption spectra in THF solution; and (**c**) energy-level diagram of porphyrins, electrolyte and TiO_2_; E_(HOMO)_ = E_oxd_ and E_(LUMO)_ = E_(HOMO)_ − E_0–0_. Adapted from reference [86] with permission from the American Chemical Society, copyright 2017.

To gain knowledge in the structure and impact of π-conjugated spacers, Krishna et al. reported a systematic study on how the introduction of auxiliary acceptors between the anchoring group and the porphyrin ring may influence the light-harvesting properties of the chromophore, the electron injection rate and the binding energy of the dye on the semiconductor surface (Figure 20) [86].

Selecting carboxylic acid as the anchoring group, these authors explored the introduction of phenyl, thiophene, furan and benzothiadiazole (either phenyl or thiophen tethered to carboxylic acid) as π-spacers. Additionally, the cyanocrylic acid group was also evaluated, linked to the porphyrin ring through phenyl or thiophen spacers. A comparison of absorption spectra showed that the presence of thiophene instead of phenyl or furan moieties resulted in a redshift of the Q band in LG2, demonstrating that the excited state was more polar than the ground state. The elongation of π-conjugation through the incorporation of a BTD moiety as an auxiliary acceptor resulted in a significant shift toward the IR region, increasing at the same time the absorption ability between the Soret and Q bands. The change of the anchoring group to cyanoacrylic acid provided broadening and redshifts in both Soret and Q bands, especially in the thiophene-tethered derivative. The photovoltaic performances were also examined, showing that the more electron-withdrawing nature of the thiophene and phenyl groups compared to the furan group resulted in better efficiency. Moreover, the presence of a thiophene instead of a phenyl linker in cyanoacrilyc-acid-anchored dyes provided a significant enhancement in the charge transfer ability and a superior DSSC performance. DFT and TD-DFT studies showed that the HOMO spread over the donor and porphyrin ring, while the LUMO spread over the porphyrin π-system and anchoring carboxylic acid groups, as known, a suitable distribution for DSSC applications.

Similar results were also reported by Lu et al., who also studied the influence of spacers on the photophysical properties of porphyrin sensitizers for solar energy conversion [87]. In this work, various π-spacers were selected, such as phenyl, thiophen, thiophene-phenyl and benzothiadiazole-phenyl, thus, including electron-deficient (such as BDT) and electron-rich (as thiophene) units. The results obtained pointed out that a single aromatic spacer improved the performance due to an enhanced electron-donating ability from phenyl to thiophene structures. However, the electron-deficient BDT aromatic unit proved to be more beneficial than the electron-rich thiophene as an extending spacer.

In order to determine the effect of the conjugation length of the π-linker in the DSSC performance, Chitpakdee et al. compared structurally related Zn porphyrins featuring a single-thiophene unit connecting an ethynylbenzoic-acid-anchoring group and a porphyrin core with the analogue displaying one more thiophene unit as the π-spacer [88]. The authors found that the dihedral angles between the aromatic porphyrin ring and the thiophene spacer were nearly perpendicular to the macrocycle in any case. However, the extension of the π-spacer with an additional thiophene unit increased these dihedral angles from 104.63° to 109.04°. Moreover, a redshift in the HOMO–LUMO energy difference was observed with the extension with one additional thiophene unit, slightly decreasing the LUMO level and increasing the HOMO level. Accordingly, experimental data on power conversion efficiency showed better performance based on the porphyrin with the extended thiophene bridge.

The incorporation of double-anchoring carboxylic acids at the β positions of monobenzoporphyrin dyes was also studied by Jinadasa et al. through the insertion of either phenyl, ethynylphenyl or vinyl spacers [89]. The results showed a significant increase in the power conversion efficiency based on the sensitizer carrying the vinyl linker, which featured a higher light-harvesting ability, a favorable HOMO/LUMO electronic distribution for electron injection and collection and a lower tendency to aggregate on the TiO_2_ surface.

**Figure 21:**
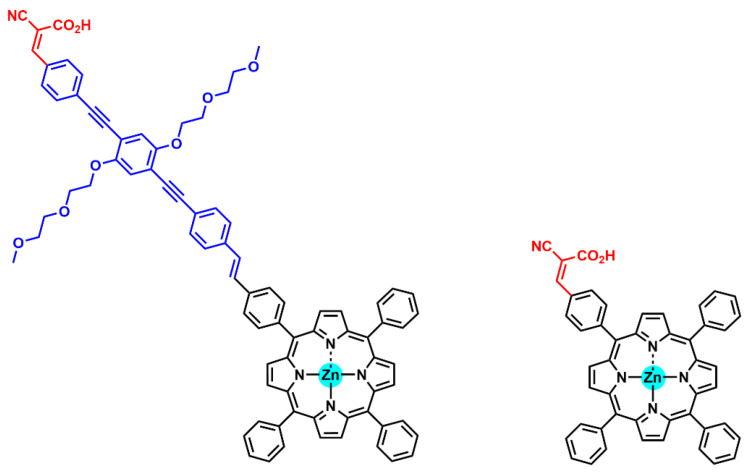
Molecular structures of porphyrin sensitizers differing in the conjugated π-spacer for improved electronic communication between dye photosensitizers and TiO_2_.

The introduction of an intramolecular dipole in the bridge was studied to assess its influence on the photovoltaic properties [90]. However, the experiments confirmed that the presence of a dipole in the spacer did not change the electronic properties of the porphyrin ring. On the other hand, Panagiotakis et al. prepared analogous donor–acceptor Zn porphyrins differentiated solely on the incorporation of a π-spacer between the porphyrin core and the cyanoacrylic-acid-anchoring group (Figure 21) [91]. Both dyes showed favorable HOMO and LUMO electronic distributions and energy levels, as well as similar absorption spectra. Noteworthy was that the dipole moment (μ) of the dye bearing the ethynyl π-spacer (13.51 D) was considerably larger than the analogue lacking the additional linker (7.40 D). These values correlated with the different power conversion efficiencies obtained with both sensitizers: 7.61% and 5.02%, respectively.

**Figure 22:**
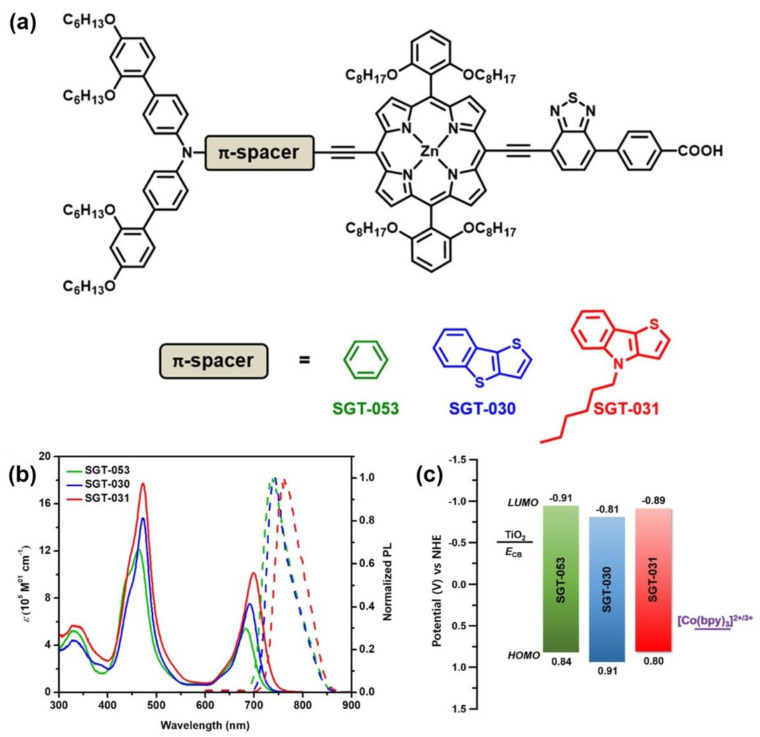
(**a**) Molecular structures of the porphyrin sensitizers featured by the incorporation of different spacers between the donor group and the porphyrin ring; (**b**) corresponding data of UV–vis absorption and emission spectra in THF solution; and (**c**) energy diagrams. SGT-053 = D–Ph–π–A; SGT-030 = D–TBT–π–A; SGT-031 = D–TI–π–A. Adapted from reference [92] with permission from the American Chemical Society, copyright 2019.

Among the strategies to extend the π-conjugation length in push–pull porphyrin structures, Ji et al. explored the introduction of conjugated spacers with differing donating abilities between the donor unit and the porphyrin core [92]. Following this idea, the authors prepared a series of D–π–A-structured Zn(II)-porphyrin dyes introducing phenyl, thieno[3,2-*b*]benzothiophene (TBT) and 4-hexyl-4H-thieno[3,2-*b*]indole (TI) moieties as the auxiliary π-spacers in the donor part of the D–π–A scaffold, featuring a benzothiadiazole acceptor structure in the acceptor part of the dye architecture (Figure 22). The influence of the extended auxiliary π-spacer was studied with spectroscopic and electrochemical studies, as well as theoretical calculations. The comparative UV–visible absorption of the three porphyrins in THF showed a redshift in the Soret and Q bands and an enhanced molar extinction coefficient for both TBT- and TI-functionalized dyes in comparison with the reference Ph-substituted porphyrin. In general, the HOMO and LUMO levels stabilized with the extension of the π-system, resulting in lower energy levels. However, the HOMO energy level of the TI-based dye presented higher energy than the other two dyes, probably due to the strong donating character of the thieno-indol moiety, resulting in a lower bandgap difference between the HOMO and the LUMO and a redshift in the absorption spectra. DFT calculations analyzing the structure of the synthesized dyes pointed out the change in the dihedral angles between the D part and the spacer moiety through the extension length of the π-spacers, resulting in significant effects on the photovoltaic properties. The dihedral angle increased with the conjugation length, which favored the charge separation, leading to an effective suppression of the charge recombination and a longer electron lifetime.

In conclusion, the introduction of a π-spacer element can have a remarkable impact on the frontier orbital energy levels. Redshifts of absorption bands are commonly observed when a π-spacer is introduced into the porphyrin architecture, which indicates a smaller energy gap between the HOMO and the LUMO and a broader absorption spectrum. This feature significantly promotes the light-harvesting ability of the sensitizer, which, ultimately, is translated into an improved photocatalytic efficiency. This strategy was successfully applied through the introduction of conjugate spacers both at the donor and acceptor regions of the porphyrin sensitizers, providing an improved electronic communication and, thus, a more efficient charge transfer.

### 2.4. Peripheral Substituents

Many lessons have been learned from the research in the field of DSSCs, where different strategies have been addressed to extend the light-harvesting ability, facilitate the charge transfer from the dye to the semiconductor and improve the durability of the photovoltaic device through the incorporation of different substituents to the aromatic core of porphyrins [41]. Among the different approaches to design efficient dye sensitizers, push–pull porphyrins following a D–π–A configuration have provided successful architectures achieving PCE records in the field of solar cells. Since the incorporation of diarylamino groups (either at the β or *meso*-positions), which provided outstanding DSSC performances [24], it was proposed that the introduction of a donor substituent would expand the absorption ability of the porphyrin and, notably, would promote the migration of excited electrons toward the acceptor or anchoring group [93]. The twisted position of the arylamino derivatives in respect to the porphyrin ring was probably responsible for the reduced charge recombination and the enhanced power conversion efficiencies [22].

The substitution at the *meso*-position was demonstrated to be more favorable in terms of the electron injection due to the electronic distribution of the porphyrin aromatic ring [41]. Substituents were introduced in the porphyrin core with the aim of extending the conjugate structure of the porphyrin ring, increase the electron stabilization capacity after photoexcitation and enhance the affinity toward the reaction substrates [26].

The most important function of peripheral substituents around the porphyrin core, either located in the β or *meso*-positions, is the ability to guide the electron transfer toward or away from the porphyrin ring. The energy levels of the porphyrin structure could be finely tuned through molecular design, varying substituents toward the correct pairing of the photosensitizer and the metal catalyst or the semiconductor [94]. Notably, substituents have a significant effect on the solubility, stability, absorption ability and electrochemical properties. HOMO and LUMO levels may be tuned through the careful selection of electron-donating and electron-withdrawing groups, also controlling the energy bandgap and, thus, the light-harvesting ability.

One of the most common problems in the use of porphyrins as photosensitizers is their natural tendency to form aggregates through π-stacking interactions, which cause the charge recombination and loss of charge transfer efficiency. To solve this problem, both bulky substituents or the further functionalization of substituents with long alkyl or alkoxyl chains have been introduced to protect the porphyrin core and reduce dye aggregation. In this line, it was recently reported that the introduction of an eight-carbon alkyl chain at the spacer of a D–π–A porphyrin sensitizer efficiently reduced dye aggregation [95]. On the downside, this had a negative impact on the dye loading, which was further reflected in a lower device performance. A similar behavior was also found when long alkoxy chains of different lengths were introduced at the *para*-position of a *meso*-phenyl group in D–π–A porphyrins [96]. In this case, a suppression in aggregates was observed, but the longer the length of the alkoxy chain, the lower the loading capacity of the dyes. On the contrary, the introduction of a methyl group at the *meta*-position in a carboxyphenyl-anchoring moiety demonstrated superior photovoltaic properties, ascribed to a lower porphyrin aggregation. Additionally, a higher dye loading was observed, which may have derived from the more vertical anchoring mode due to the steric hindrance associated with the methyl group. All these systematic studies suggest that the introduction of bulkiness in the proximity of the porphyrin core can have a remarkable impact on the photovoltaic performance, so the position, number and length of bulky moieties should be carefully designed to obtain improved efficiencies [97,98].

**Figure 23:**
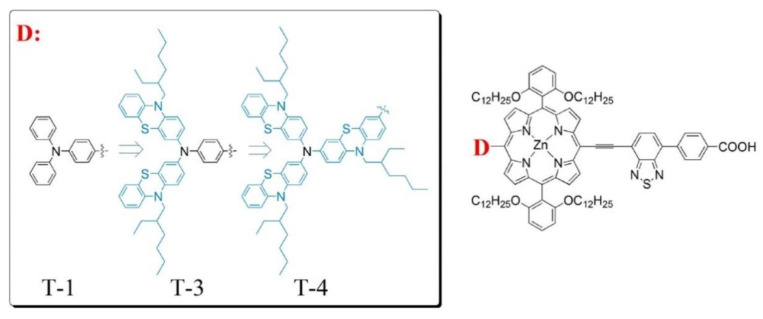
Molecular structures of porphyrin dyes showing increasingly bulky groups at the donor substituent. Reproduced from reference [99] with permission from the American Chemical Society, copyright 2021.

The use of diarylamino groups as the “push” element in D–π–A porphyrin sensitizers together with the remarkable influence on photovoltaic performance triggered an intense activity to determine the effect of the number and the position of these substituents and search for alternative electron-donating groups (EDG) as replacements. In this field, Li et al. recently proposed the use of phenothiazine donor groups instead of benzene units in the original triphenylamine group (Figure 23) [99].

**Figure 24:**
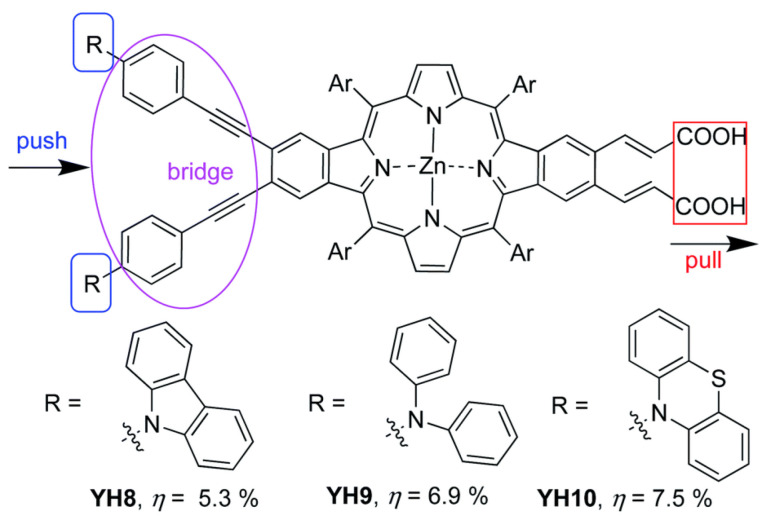
Molecular structures of the porphyrin dyes for the investigation of the push group effect on β-functionalized push–pull dibenzoporphyrins. Adapted from reference [101] with permission from the Royal Society of Chemistry, copyright 2021.

In this work, a remarkable enhancement in the PCE and an excellent electron injection ability for the corresponding photovoltaic device were obtained, especially in the case of T-3, where two phenothiazine units were incorporated into the donor amine moiety. DFT studies have shown that the presence of these units in the D–π–A porphyrin shifted the HOMO distribution toward the D unit, while the HOMO of the triphenylamine substituted dye, T-1, was extended over the donor unit and the porphyrin macrocycle. This change in distribution probably arose from the steric volume of the phenothiazine donor moieties, resulting in larger dihedral angles in respect to the macrocycle and, hence, in a better charge separation. These results were also confirmed by other authors who used phenothiazine derivatives as strong donor substituents [100,101,102]. Along these lines, Hu et al. prepared a series of β-functionalized push–pull dibenzoporphyrins with different arylamine substituents as the donor groups (Figure 24) [101]. The best performances observed corresponded to the phenothiazine YH10 and diphenylamine YH9 derivatives, which correlated with a well-differentiated HOMO/LUMO distribution that boosted the electron injection and charge separation. These features resulted in a lower charge recombination and a higher efficiency of the corresponding DSSCs. In contrast, when carbazole was installed as the donor group YH8, the push–pull effect was neutralized, and no implication of the carbazole was observed in the HOMO energy level distribution. This feature led to a lower PCE for YH8-based DSSCs.

**Figure 25:**
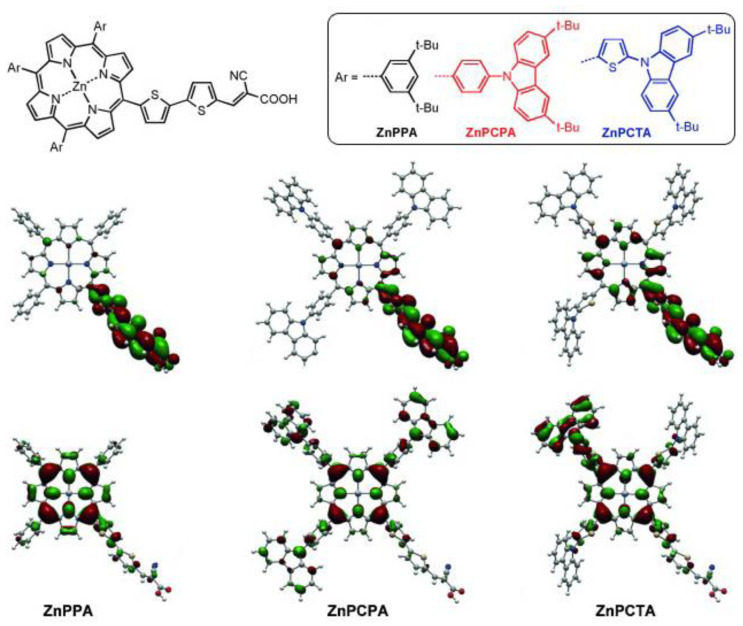
Electronic distribution of the frontier molecular orbitals (HOMO and LUMO) computed at the B3LYP/6-31G(d,p) level. Adapted from reference [103] with permission from Wiley, copyright 2015.

Regarding this matter, *meso*-substituted A_3_B porphyrins functionalized with three carbazole phenyl groups were studied (Figure 25) [103]. The presence of an additional aryl unit between the carbazole and the porphyrin core (ZnPCPA and ZnPCTA) had a remarkable impact on the charge separation. The computational analysis of the frontier orbitals distribution showed a HOMO with π-electrons delocalized both on the porphyrin ring and the carbazole substituent for ZnPCPA and ZnPCTA, while the HOMO of the ZnPPA sensitizer showed a more localized electronic distribution, mainly at the porphyrin macrocycle. This difference was, therefore, translated into a better charge separation ability and a higher current conversion efficiency.

**Figure 26:**
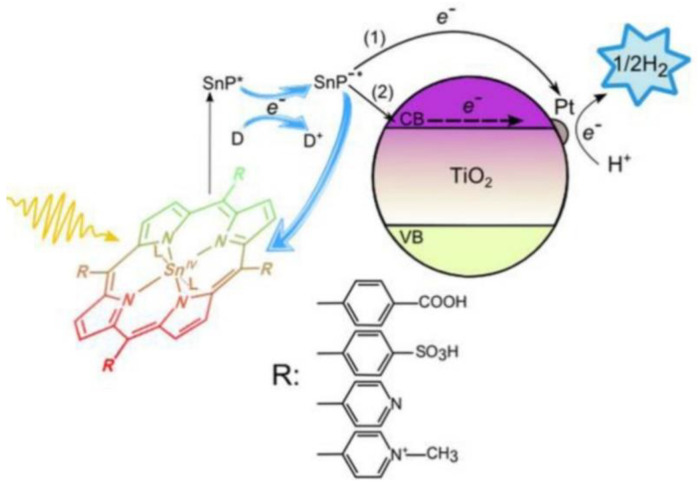
Schematic representation of the photoexcitation and electron transfer processes for hydrogen evolution under visible light irradiation in the presence of Sn(IV) porphyrins/Pt-TiO_2_ nanocomposites. Reproduced from reference [94] with permission from the American Chemical Society, copyright 2016.

In the context of photochemical catalysis, Koposova et al. studied the relationship between the structure of the *meso*-substituents of a series of tin(IV) porphyrins and their ability as sensitizers for the photocatalyzed hydrogen evolution [94]. Making use of Sn(IV)-porphyrins/Pt-TiO_2_ nanocomposite systems, the authors examined the photocatalytic performance of water-soluble porphyrins. Sn(IV) was selected as the central cation due to its strong electronegativity, able to pull the electron density of the porphyrin to the core and, thus, being capable of stabilizing the product of a one-electron reduction (a π-radical anion) for minutes at a neutral pH or even for hours in alkaline solutions. This feature makes Sn(IV) porphyrins good candidates for the design of photocatalytic systems for H_2_ generation. As a result, the long-lived reduced species of Sn(IV) porphyrins would avoid the need for close contact or a suitable anchoring group between the sensitizer and the semiconductor. In this work, *meso*-tetra-aryl-substituted Sn(IV) porphyrins were analyzed, featuring either 4-sulfonatophenyl, 4-carboxyphenyl, 4-pyridyl or *N*-methyl-4-pyridyl groups in the periphery of the Sn porphyrin (Figure 26). All these groups are electron-withdrawing substituents, which favored the reduction in the porphyrin in the ground state, making it easily reducible with the use of the EDTA in the excited state to produce the corresponding π-radical anions. The derivatives showing more positive reduction potentials, corresponding to the porphyrins with more positive net ring charge (such as the porphyrins substituted with *N*-methyl-4-pyridyl or 4-pyridyl) produced long-lived π-radical anions and favored the formation of phlorin species, a key intermediate involved in H_2_ production.

Although *N*-methyl-4-pyridyl showed the most favorable photoredox properties, the steric hindrance due to the presence of the methyl group at the pyridyl ring prevents the close contact between the porphyrin and the TiO_2_ semiconductor or the Pt surface, hampering the crucial electron transfer for photocatalysis activity. Porphyrins with lower electron-withdrawing properties and, therefore, with a more negative reduction potential, such as those decorated with 4-sulfonatophenyl and 4-carboxyphenyl, showed poorer hydrogen generation. As a conclusion, the incorporation of electron-withdrawing substituents adequately tune the photoredox properties of Sn(IV) porphyrins to easily produce π-radical anions, which are key intermediates in the production of H_2_. Additionally, steric factors also play a role, determining the ability of these porphyrins to bind to the active catalytic surface and allow for the necessary electron transfer in the photocatalyst.

The influence of the substituents was also studied by Liu et al. in the fabrication of nano-biosensors to detect cholesterol in human serum based on peroxidase-like activity [104]. In this work, hollow TiO_2_ nanospheres self-doped with Ti^3+^ (so-called b-TiO_2_) were selected as the active photocatalytic surfaces, where free-base-substituted porphyrins were attached through hydrogen bonds and electrostatic interactions.

**Figure 27:**
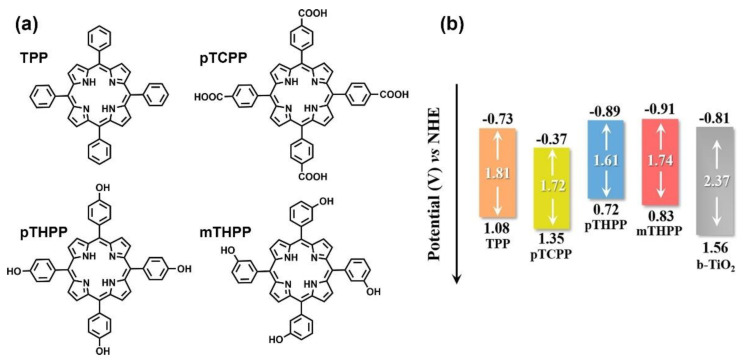
(**a**) Chemical structures of the porphyrin studied: *meso*-tetrakis(phenyl) porphyrin (TPP), *meso*-tetrakis(4-carboxylphenyl) porphyrin (pTCPP), *meso*-tetrakis(4-(hydroxyl)phenyl) porphyrin (*p*THPP) and *meso*-tetrakis(3-(hydroxyl)phenyl) porphyrin (*m*THPP); (**b**) comparison of band structure of b-TiO_2_ and the four porphyrins. Adapted from reference [104] with permission from Elsevier, copyright 2022.

**Figure 28:**
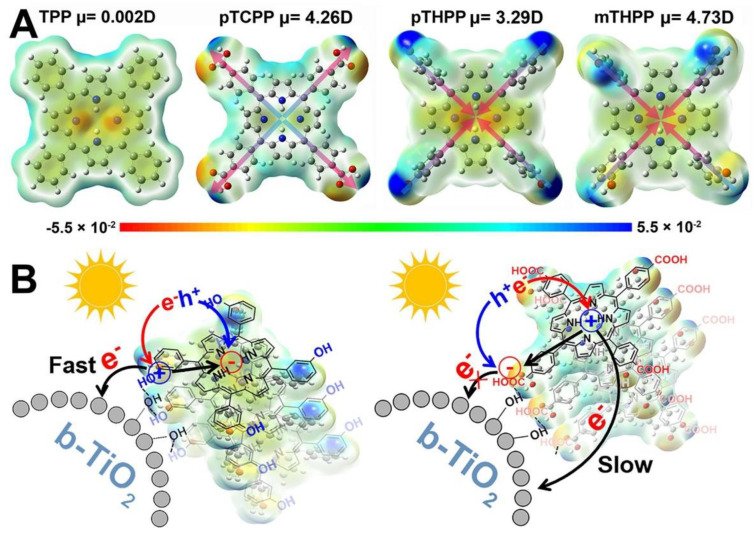
(**A**) Electron distribution and molecular dipoles in the four porphyrins studied (the direction of the arrow represents the direction of the built-in electric field); (**B**) illustration of charge transfer for b-TiO_2_/29mTHPP (left) and b-TiO_2_/29pTCPP (right) under light. Reproduced from reference [104] with permission from Elsevier, copyright 2022.

A series of porphyrins decorated with substituents featuring different electronegativity moieties at the *meso*-position were tested (Figure 27a): *meso*-tetrakis(phenyl) porphyrin (TPP), *meso*-tetrakis(4-carboxylphenyl) porphyrin (pTCPP); *meso*-tetrakis(4-(hydroxyl)phenyl) porphyrin (*p*THPP) and *meso*-tetrakis(3-(hydroxyl)phenyl) porphyrin (*m*THPP). The energy levels of the HOMO and the LUMO were remarkably influenced by the substitution. Thus, electron-donating groups increased the HOMO and LUMO levels, while electron-withdrawing groups had the opposite effect (Figure 27b). As a result, the presence of the hydroxyl groups provided the most favorable distribution of orbitals, hence, effectively promoting the charge transfer from the sensitizer to the active surface. These porphyrins also showed the largest dipole moments built in the porphyrin ring (Figure 28A). The authors reasoned that this large dipole favored the formation of an electric field that promoted the outward migration of photogenerated electrons to the periphery of the porphyrins (Figure 28B). Accordingly, the subsequent electron transfer to the semiconductor was promoted, and the light-induced POD-like catalytic activity was enhanced.

An alternative mode of substitution was explored by Nikolaou et al., constructing Zn porphyrins functionalized both at the *meso*- and axial-coordinated positions with BODIPY substituents [105]. The photocatalytic system developed in this work consisted of Zn porphyrins chemisorbed onto Pt-TiO_2_ nanoparticles. The addition of a BODIPY unit increased the absorption spectrum of the sensitizer between 450 and 550 nm. This was precisely the region in which the porphyrin hardly absorbed, thus, increasing the efficiency of light-harvesting and photocatalytic activity. The authors demonstrated that the addition of an extra unit of BODIPY chromophore had a positive impact on the photocatalytic H_2_ evolution, leading to enhanced TON values. However, the incorporation of a single BODIPY unit covalently attached to the porphyrin ring showed a much greater efficiency in comparison to the axially coordinated chromophore via the imidazole linkage. These results emphasize the importance of the direct electron injection via a covalent bond to the porphyrin core in contrast to the moderate antenna effect of substituents axially coordinated through the central metal.

**Figure 29:**
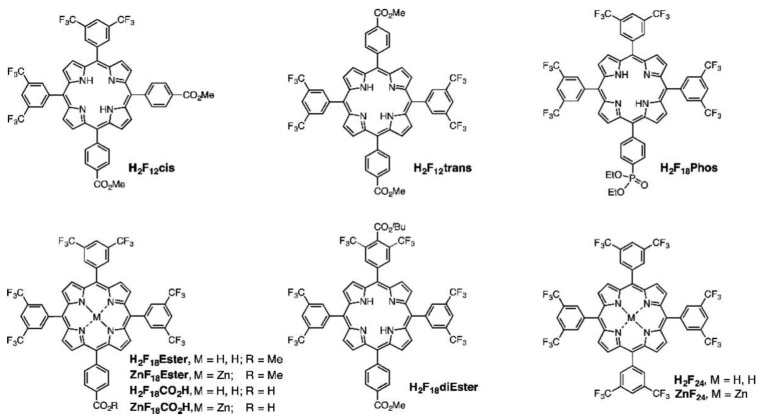
Structures of ten trifluoromethylated porphyrins. Reproduced from reference [106] with permission from the American Chemical Society, copyright 2016.

In the context of dye-sensitized photoelectrochemical water splitting, a photoanode is constructed based on mesoporous semiconductors functionalized with dye sensitizers and water oxidation catalysts. Upon light absorption, the chromophore injects the photogenerated electrons into the CB of the semiconductor. These electrons migrate through an external circuit to the cathode where the proton reductions take place to produce hydrogen. The positive charge generated in the dye sensitizer (the hole) is quenched by the activity of the coupled water oxidation catalysts, which are responsible for the oxidation of water to oxygen. In order to work as efficient sensitizer, the energy levels of the HOMO and the LUMO should be aligned with the redox potentials of both the water oxidation catalyst and the semiconductor, respectively. Thus, more positive potentials than that of the catalyst are needed to facilitate oxidation. Keeping these requirements in mind, Jiang et al. proposed the use of porphyrins substituted at the *meso*-positions with electron-withdrawing groups to decrease the energy of the frontier orbitals and obtain more positive potentials to allow for water oxidation [106]. Commonly, pentafluorophenyl groups are used as *meso*-substituents [107], but stability problems may arise from their sensitivity to nucleophilic aromatic substitution at the *para* C-F position. Thus, an alternative strategy based on the use of trifluoromethyl moieties was proposed, and a systematic study was carried out by increasing the number of bis(3,5-trifluoromethyl)phenyl groups incorporated into the *meso*-positions (Figure 29). The results obtained indicated that only the metallated species ZnF_18_Ester and ZnF_24_ were able to inject electrons into the CB of TiO_2_. Notably, all the porphyrins prepared showed a highly oxidizing potential, and from 12 to 18 fluorine atoms in the series, more positive values correlated with the increasing number of phenyl-CF_3_ groups. These results demonstrate that the incorporation of CF_3_ substituents at the *meso*-position of porphyrins constitute a practical strategy to modulate oxidative potentials compatible with water oxidation catalysts.

As a conclusion, the substitution of the porphyrin macrocycle offers a valuable tool for tuning important features of the resulting sensitizers, such as light absorbance, charge injection ability, aggregation bias and solubility. Through the incorporation of different substituents, it is possible to adjust the energy of the frontier orbitals and, hence, the photoredox capacity of the dye, which is key for an effective charge transfer to the semiconductor. An elongated D–π–A architecture also promotes charge separation. Thus, the incorporation of donor groups in an opposite position to the anchoring moiety effectively distributes the LUMO around the electron-withdrawing anchoring moiety, while the HOMO is shifted to the porphyrin macrocycle and the donor group. This distribution contributes to longer excited-state lifetimes and a lower charge recombination. Notably, when using a sacrificial electron donor, substitutions at the porphyrin core may be selected to increase the interaction with the SED species. With this aim, both the energy levels and polarity should be considered, hence, providing a more efficient regeneration of the active dye.

### 2.5. Ring Core

**Figure 30:**
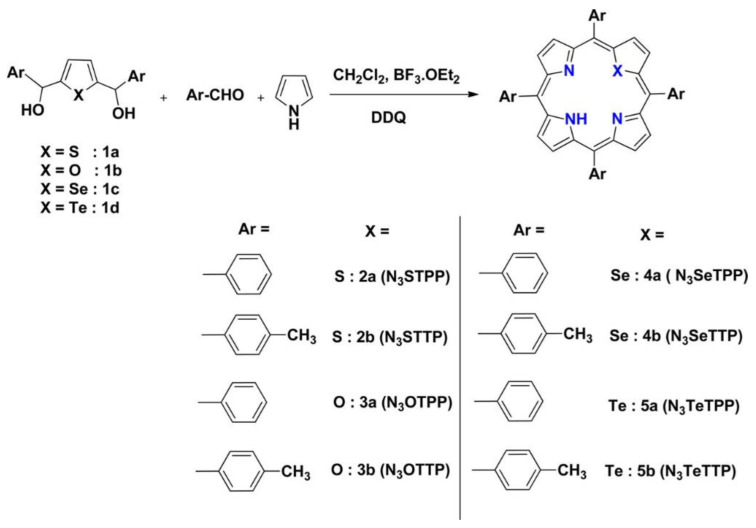
Synthesis of symmetrical 21-monoheteroatom-substituted porphyrins. Reproduced from reference [110] with permission from the American Chemical Society, copyright 2017.

Core-modified porphyrins stemming from the replacement of one or two of their pyrrole nitrogen’s through different heteroatoms constitute an interesting class of porphyrin analogues (Figure 30) [108]. This is due to the fact that their physicochemical features greatly differ from their N4-containing counterparts [109]. Along these lines, the incorporation of bulky atoms into the porphyrin core may perturb the planar structure of the ring through steric factors. This structural distortion can alter the redox potential of the heteroporphyrin and, as a result, both the optical properties and the photocatalytic activity of the dye-sensitized semiconductor [51]. In this regard, when taking their azaporphyrinic equivalents as a reference, heteroporphyrins exhibit remarkable bathochromic shifts in the Soret and Q bands. In this case, the magnitude of this effect is dependent on the number and size of heteroatoms incorporated into the porphyrin core [110]. Thus, this redshift is larger for di-heteroatom-substituted porphyrins than for those monosubstituted, while for a given number of substitutions, the bathochromic shift becomes more significant as the size of the heteroatom increases. These optical attributes may render these organic dyes as valuable photosensitizers given their ability to extend the photoresponse of the semiconductor to the near-infrared region in the search for a better exploitation of the full solar light spectrum [111]. Ring deformations can also lead to a change in the coordination abilities of heteroporphyrins toward metal ions. At this point, it should be noted that these dyes can coordinate with atoms in atypical oxidation states, such as Cu^+^ or Ni^+^, which is rarely found when it comes to N4 porphyrins [112]. In a similar way, novel affinities of the metal center for axial ligands may also be found when studying metallated heteroporphyrins. Nevertheless, while these molecules have been widely explored from a synthetic point of view [110], their utilization in photocatalysis is still lacking [113]. Several factors may lie behind the limited scope of heteroporphyrins in energy-related research. In this respect, poor quantum yields and low singlet excited-state lifetimes have usually been reported for these compounds [110]. As known, these attributes are detrimental to photocatalytic performance in terms of electron–hole recombination. Additionally, heteroporphyrins may be difficult to oxidize (easy to reduce) compared to regular azaporphyrins [109]. This redox behavior stems from the electron-withdrawing effect exerted by the heteroatoms on the frontier orbital of these dyes, somehow defying the very operational principles governing the photocatalytic process in architectures where the dye is expected to efficiently inject electrons into the conduction band of the semiconductor.

There have only been a few reports on the implementation of heteroporphyrins in light-harvesting nanomaterials based on semiconductors and, as is often the case, photovoltaic studies seem to have led the way. Xie et al. described the application of nine mono- and dithiaporphyrins as sensitizers in DSSCs [114]. In this case, the device efficiencies were found to be as high as 0.19%. Similarly, through a systematic structural modification, Mane et al. synthesized a series of mono- and dicarboxylate-functionalized A_3_B and A_2_B_2_ thiaporphyrins [115]. Through this design, the best cell performance was given by an overall photon-to-current conversion efficiency of 1.69%. However, no direct comparisons with their N4-containing counterparts were established in these works. Mane et al. addressed this issue by applying as sensitizing dyes a free-base regular porphyrin (N4), a thiaporphyrin (N3S) and an oxaporphyrin (N3O) [116]. In this context, the overall efficiencies of the built solar cells were in the order N4 (3.66%) >> N3S (0.22%) > N3O (0.01%). The fact that the cells integrating regular azaporphyrins outperformed those devices incorporating heteroporphyrins was ascribed to the poor electron injection resulting from the rapid excited-state relaxation, as evidenced by the fluorescence lifetimes. These authors also reported on the use of expanded heteroporphyrins as DSSC sensitizers [117]. In this case, panchromatic incident photon-to-current efficiencies, high short-circuit photocurrent densities and good overall efficiencies were found, revealing the potential of expanded core-modified porphyrins in solar cell applications. For the same purpose, Vijay et al. assessed the suitability of different oxaporphyrins and thiaporphyrins through density functional theory calculations [118]. As seen in this work, the HOMO–LUMO gaps were found to be lower than many of their N4-contaning counterparts, with adsorption spectra extending well into the near infrared region. Likewise, the simulated density of states pointed out to a weak electronic coupling between the dyes and TiO_2_, which favored electron injection from the excited state of the dye to the conduction band of this semiconductor. It is also worth noting that the introduction of heteroatoms in the porphyrin core may have a direct impact on the affinity of these chromophores for TiO_2_ surfaces. Along these lines, oxygen-substituted cores constitute an electron-rich environment, which might decrease the acidity of peripheral carboxylic groups acting as anchoring points, thus, diminishing the affinity of oxaporphyrins for metal oxide semiconductors [116].

Besides heteroatom replacements, the synthetic design of the porphyrin core has also been extended to modifications in the cavity size and shape of these macrocycles [119]. In this context, expanded porphyrins have received considerable attention due to their critical role in the study of fundamental questions related to aromaticity [120]. In this line, these compounds are well-known for reversibly affording counterpart macrocycles featuring [4n + 2] and [4n] π electrons (aromatic and antiaromatic, respectively) by means of different strategies [121]. For this reason, expanded porphyrins have become exceptional testbeds for assessing the relationships between aromaticity and photophysical properties [122]. This is particularly interesting when it comes to photocatalytic applications, as previous studies have shown that aromatic expanded porphyrins display more intense Soret and Q bands compared to those found for their antiaromatic homologues [123]. In the same vein, other works have proved excited-state dynamics to be dependent on aromaticity [124]. In this case, longer excited-state lifetimes were found to be associated with molecular structures showing aromatic behavior. In view of these results, it seems that aromaticity should be considered when designing efficient sensitizers, although its multifaceted nature in porphyrinoids still renders its quantification a major challenge [125].

Taking all the above into account, it is clear that the implementation of core-modified porphyrins in TiO_2_ photocatalysis should exploit the remarkable light-harvesting properties of these engineered molecules, while improving those physicochemical features that may potentially hinder their use as electron-injecting antennas. This should be accomplished by means of a rational and integral design of the porphyrin structure, in which, through a cooperative effort, metals, anchoring groups, spacers and peripheral substituents boost the photoactivity of the dye as a whole. Finally, it is important to stress that heteroporphyrins may constitute excellent alternatives to N4 porphyrins in chemical transformations where metals with unusual oxidation states are required, or a higher degree of exposure of the metal center is needed. Regarding the latter, the distorting effect arising from the size and position of the metal center in combination with the sterically overloaded core of the heteroporphyrin is expected to promote nonplanar architectures, thus, allowing guest molecules to directly interact with the exposed cores. In the end, providing reactive molecules with an efficient way to approach, bind and release from the catalytically active sites is key for the design of high-performance nanocatalysts.

## 3. Conclusions

Great efforts have been devoted over recent years in order to design novel porphyrin architectures aiming to improve light-harvesting capabilities, redox potentials and electron injection properties to efficiently collect energy from sunlight. In this review, the key structural motifs of these chromophores were examined, placing a particular emphasis on dye-sensitized photocatalysts. First, these architectural elements should be adequately designed to be compatible with the reaction conditions under which DSPs operate. Concerning stability, robust bidentate or tridentate anchoring groups should be selected, capable of ensuring a reliable connection with the TiO_2_ surface, even in acidic or basic solutions. Additionally, the photocatalytic efficiency is strongly determined by both the central metal and the substituents at the periphery of the macrocycle. Thus, the central metal can have a big impact on the redox potential of the sensitizer as well as the coordination ability with reactants or other porphyrin units. At the same time, substituents should promote charge transfer through an internal dipole based on an asymmetric electronic distribution between the donor and the acceptor groups in a D–π–A configuration. Further elements, such as bulky groups or long alkoxyl chains, could be introduced to harness undesirable porphyrin aggregation. Notably, substituents can also tune the affinity of the sensitizer for the SED, thus, boosting dye regeneration. Supplementary π-spacers can aid the expansion of the electronic distribution, reducing the bandgap between the HOMO and the LUMO and enhancing light-harvesting abilities. The distortion of the ring core through the preparation of heteroporphyrins can also provide additional opportunities to chelate atypical oxidation states of metals, such as Cu^+^ or Ni^+^, thus, opening the door to novel catalytic processes. Further synthetic developments are expected in the near future, aiming to expand the remarkable efficiency of these chromophores as both light antennas and active catalytic centers. Along these lines, the incorporation of porphyrin derivatives to semiconductor/cocatalyst systems, or their combination with noble metal nanoparticles in carefully engineered nanostructures, is envisaged to provide more definable and enhanced performances in DSP composites. Furthermore, by taking advantage of polymeric materials such as MOFs, COFs or HOFs, it is foreseeable that novel molecular designs will arise in order to boost the potential of these porous structures in challenging chemical transformations, such as CO_2_ reduction reactions or nitrogen fixation processes.

## Data Availability

Not applicable.

## References

[1] Hammarström L., Hammes-Schiffer S. (2009). Artificial Photosynthesis and Solar Fuels. Acc. Chem. Res..

[2] Hisatomi T., Domen K. (2019). Reaction Systems for Solar Hydrogen Production via Water Splitting with Particulate Semiconductor Photocatalysts. Nat. Catal..

[3] Lin S., Huang H., Ma T., Zhang Y. (2021). Photocatalytic Oxygen Evolution from Water Splitting. Adv. Sci..

[4] Yao S., He J., Gao F., Wang H., Lin J., Bai Y., Fang J., Zhu F., Huang F., Wang M. (2023). Highly Selective Semiconductor Photocatalysis for CO_2_ Reduction. J. Mater. Chem. A.

[5] Hong J., Cho K.-H., Presser V., Su X. (2022). Recent Advances in Wastewater Treatment Using Semiconductor Photocatalysts. Curr. Opin. Green Sustain. Chem..

[6] Zhang G., Sewell C.D., Zhang P., Mi H., Lin Z. (2020). Nanostructured Photocatalysts for Nitrogen Fixation. Nano Energy.

[7] Schneider J., Matsuoka M., Takeuchi M., Zhang J., Horiuchi Y., Anpo M., Bahnemann D.W. (2014). Understanding TiO_2_ Photocatalysis: Mechanisms and Materials. Chem. Rev..

[8] Dong H., Zeng G., Tang L., Fan C., Zhang C., He X., He Y. (2015). An Overview on Limitations of TiO_2_-Based Particles for Photocatalytic Degradation of Organic Pollutants and the Corresponding Countermeasures. Water Res..

[9] Abe R. (2010). Recent Progress on Photocatalytic and Photoelectrochemical Water Splitting under Visible Light Irradiation. J. Photochem. Photobiol. C Photochem. Rev..

[10] Guo Q., Zhou C., Ma Z., Yang X. (2019). Fundamentals of TiO_2_ Photocatalysis: Concepts, Mechanisms, and Challenges. Adv. Mater..

[11] Sulaiman S.N.A., Zaky Noh M., Nadia Adnan N., Bidin N., Ab Razak S.N. (2018). Effects of Photocatalytic Activity of Metal and Non-Metal Doped TiO_2_ for Hydrogen Production Enhancement—A Review. J. Phys. Conf. Ser..

[12] Liu Z., Yu Y., Zhu X., Fang J., Xu W., Hu X., Li R., Yao L., Qin J., Fang Z. (2022). Semiconductor Heterojunctions for Photocatalytic Hydrogen Production and Cr(VI) Reduction: A Review. Mater. Res. Bull..

[13] Niu B., Wang X., Wu K., He X., Zhang R. (2018). Mesoporous Titanium Dioxide: Synthesis and Applications in Photocatalysis, Energy and Biology. Materials.

[14] Zhang X., Peng T., Song S. (2016). Recent Advances in Dye-Sensitized Semiconductor Systems for Photocatalytic Hydrogen Production. J. Mater. Chem. A.

[15] O’Regan B., Grätzel M. (1991). A Low-Cost, High-Efficiency Solar Cell Based on Dye-Sensitized Colloidal TiO_2_ Films. Nature.

[16] Ren Y., Zhang D., Suo J., Cao Y., Eickemeyer F.T., Vlachopoulos N., Zakeeruddin S.M., Hagfeldt A., Grätzel M. (2023). Hydroxamic Acid Preadsorption Raises Efficiency of Cosensitized Solar Cells. Nature.

[17] Pellegrin Y., Odobel F. (2017). Sacrificial Electron Donor Reagents for Solar Fuel Production. Comptes Rendus Chim..

[18] Zani L., Melchionna M., Montini T., Fornasiero P. (2021). Design of Dye-Sensitized TiO_2_ Materials for Photocatalytic Hydrogen Production: Light and Shadow. J. Phys. Energy.

[19] Tomar N., Agrawal A., Dhaka V.S., Surolia P.K. (2020). Ruthenium Complexes Based Dye Sensitized Solar Cells: Fundamentals and Research Trends. Sol. Energy.

[20] Légalité F., Escudero D., Pellegrin Y., Blart E., Jacquemin D., Fabrice O. (2019). “Iridium Effect” in Cyclometalated Iridium Complexes for p-Type Dye Sensitized Solar Cells. Dye. Pigment..

[21] Błaszczyk A. (2018). Strategies to Improve the Performance of Metal-Free Dye-Sensitized Solar Cells. Dye. Pigment..

[22] Ramasamy S., Bhagavathiachari M., Suthanthiraraj S.A., Pichai M. (2022). Mini Review on the Molecular Engineering of Photosensitizer: Current Status and Prospects of Metal-Free/Porphyrin Frameworks at the Interface of Dye-Sensitized Solar Cells. Dye. Pigment..

[23] Youssef Z., Colombeau L., Yesmurzayeva N., Baros F., Vanderesse R., Hamieh T., Toufaily J., Frochot C., Roques-Carmes T. (2018). Dye-Sensitized Nanoparticles for Heterogeneous Photocatalysis: Cases Studies with TiO_2_, ZnO, Fullerene and Graphene for Water Purification. Dye. Pigment..

[24] Urbani M., Grätzel M., Nazeeruddin M.K., Torres T. (2014). Meso-Substituted Porphyrins for Dye-Sensitized Solar Cells. Chem. Rev..

[25] Urbani M., Ragoussi M.-E., Nazeeruddin M.K., Torres T. (2019). Phthalocyanines for Dye-Sensitized Solar Cells. Coord. Chem. Rev..

[26] Zhang Y., Ren K., Wang L., Wang L., Fan Z. (2022). Porphyrin-Based Heterogeneous Photocatalysts for Solar Energy Conversion. Chin. Chem. Lett..

[27] O’Neill J.S., Kearney L., Brandon M.P., Pryce M.T. (2022). Design Components of Porphyrin-Based Photocatalytic Hydrogen Evolution Systems: A Review. Coord. Chem. Rev..

[28] Li A., Chen S., Yang F., Gao H., Dong C., Wang G. (2021). Metalloporphyrin-Decorated Titanium Dioxide Nanosheets for Efficient Photocatalytic Carbon Dioxide Reduction. Inorg. Chem..

[29] Wang L., Huang G., Zhang L., Lian R., Huang J., She H., Liu C., Wang Q. (2022). Construction of TiO_2_-Covalent Organic Framework Z-Scheme Hybrid through Coordination Bond for Photocatalytic CO_2_ Conversion. J. Energy Chem..

[30] Wang Y., Zhao Z., Sun R., Bian J., Zhang Z., Jing L. (2022). TiO_2_-Modulated Tetra(4-Carboxyphenyl)Porphyrin/Perylene Diimide Organic Z-Scheme Nano-Heterojunctions for Efficient Visible-Light Catalytic CO_2_ Reduction. Nanoscale.

[31] Liu H., Chen S., Zhang Y., Li R., Zhang J., Peng T. (2022). An Effective Z-Scheme Hybrid Photocatalyst Based on Zinc Porphyrin Derivative and Anatase Titanium Dioxide Microsphere for Carbon Dioxide Reduction. Mater. Today Sustain..

[32] Ma Y., Yi X., Wang S., Li T., Tan B., Chen C., Majima T., Waclawik E.R., Zhu H., Wang J. (2022). Selective Photocatalytic CO_2_ Reduction in Aerobic Environment by Microporous Pd-Porphyrin-Based Polymers Coated Hollow TiO_2_. Nat. Commun..

[33] Yang T.-Y., Zhang Y., Zhang G.-L., Zhang J.-J., Zhang Y.-H. (2023). A Sulfonated Porphyrin Polymer/P25m Composite for Highly Selective Photocatalytic Conversion of CO_2_ into CH_4_. Catal. Lett..

[34] Xiao T., Chen Y., Liang Y. (2022). Ni(II) Tetra(4-Carboxylphenyl)Porphyrin-Sensitized TiO_2_ Nanotube Array Composite for Efficient Photocatalytic Reduction of CO_2_. J. Phys. Chem. C.

[35] Xiao T., Chen Y., Liang Y. (2022). Visible Light Responsive Metalloporphyrin-Sensitized TiO_2_ Nanotube Arrays for Artificial Photosynthesis of Methane. React. Chem. Eng..

[36] Zhang C., Yang J., Hara K., Ishii R., Zhang H., Itoi T., Izumi Y. (2022). Anchoring and Reactivation of Single-Site Co–Porphyrin over TiO_2_ for the Efficient Photocatalytic CO_2_ Reduction. J. Catal..

[37] Wu H., Tan H.L., Toe C.Y., Scott J., Wang L., Amal R., Ng Y.H. (2020). Photocatalytic and Photoelectrochemical Systems: Similarities and Differences. Adv. Mater..

[38] Yang J., Jing J., Zhu Y. (2021). A Full-Spectrum Porphyrin–Fullerene D–A Supramolecular Photocatalyst with Giant Built-In Electric Field for Efficient Hydrogen Production. Adv. Mater..

[39] Nasrollahi R., Martín-Gomis L., Fernández-Lázaro F., Zakavi S., Sastre-Santos Á. (2019). Effect of the Number of Anchoring and Electron-Donating Groups on the Efficiency of Free-Base- and Zn-Porphyrin-Sensitized Solar Cells. Materials.

[40] Chen Y., Mo Z., Zhu X., Xu Q., Xue Z., Li H., Xu H., Zhao L. (2021). Facilitated Interfacial Charge Separation Using Triphenylamine-Zinc Porphyrin Dyad-Sensitized TiO_2_ Nanoparticles for Photocatalysis. J. Alloys Compd..

[41] Li L.L., Diau E.W.G. (2013). Porphyrin-Sensitized Solar Cells. Chem. Soc. Rev..

[42] Ji J.M., Zhou H., Kim H.K. (2018). Rational Design Criteria for D-π-A Structured Organic and Porphyrin Sensitizers for Highly Efficient Dye-Sensitized Solar Cells. J. Mater. Chem. A.

[43] Zeng K., Tong Z., Ma L., Zhu W.H., Wu W., Xie Y., Xie Y. (2020). Molecular Engineering Strategies for Fabricating Efficient Porphyrin-Based Dye-Sensitized Solar Cells. Energy Environ. Sci..

[44] Lu J., Liu S., Wang M. (2018). Push-Pull Zinc Porphyrins as Light-Harvesters for Efficient Dye-Sensitized Solar Cells. Front. Chem..

[45] Higashino T., Imahori H. (2015). Porphyrins as Excellent Dyes for Dye-Sensitized Solar Cells: Recent Developments and Insights. Dalton Trans..

[46] Shalini S., Balasundaraprabhu R., Kumar T.S., Prabavathy N., Senthilarasu S., Prasanna S. (2016). Status and Outlook of Sensitizers/Dyes Used in Dye Sensitized Solar Cells (DSSC): A Review. Int. J. Energy Res..

[47] Birel Ö., Nadeem S., Duman H. (2017). Porphyrin-Based Dye-Sensitized Solar Cells (DSSCs): A Review. J. Fluoresc..

[48] Gomes C., Peixoto M., Pineiro M. (2021). Modern Methods for the Sustainable Synthesis of Metalloporphyrins. Molecules.

[49] Yang L., Cheng Y., Fan D., Li Z. (2022). Recent Research Progress and Perspectives on Porphyrin-Based Porous Photocatalysts in the Field of CO_2_ Reduction. Energy Fuels.

[50] Chanhom P., Charoenlap N., Manipuntee C., Insin N. (2019). Metalloporphyrins-Sensitized Titania-Silica-Iron Oxide Nanocomposites with High Photocatalytic and Bactericidal Activities under Visible Light Irradiation. J. Magn. Magn. Mater..

[51] Valicsek Z., Horváth O. (2013). Application of the Electronic Spectra of Porphyrins for Analytical Purposes: The Effects of Metal Ions and Structural Distortions. Microchem. J..

[52] Zhao X., Liu X., Yu M., Wang C., Li J. (2017). The Highly Efficient and Stable Cu, Co, Zn-Porphyrin–TiO_2_ Photocatalysts with Heterojunction by Using Fashioned One-Step Method. Dye. Pigment..

[53] Chen Y., Wang M., Yan F., Wang W., Qu Y., Fang H., Cui Z., He B., Li J. (2022). Enhanced UV–Vis Photoinduced Hydrogen Evolution of Metalloporphyrin Sensitized PSf/TiO_2_ MMMs by Varying Center Metal Ion Complexed in Porphyrin. Fuel.

[54] Arkan F., Izadyar M. (2017). The Investigation of the Central Metal Effects on the Porphyrin-Based DSSCs Performance; Molecular Approach. Mater. Chem. Phys..

[55] Huang L.-Y., Huang J.-F., Lei Y., Qin S., Liu J.-M. (2020). Porous Hybrid Materials Based on Mesotetrakis(Hydroxyphenyl) Porphyrins and TiO_2_ for Efficient Visible-Light-Driven Hydrogen Production. Catalysts.

[56] Nikolaou V., Charalambidis G., Ladomenou K., Nikoloudakis E., Drivas C., Vamvasakis I., Panagiotakis S., Landrou G., Agapaki E., Stangel C. (2021). Controlling Solar Hydrogen Production by Organizing Porphyrins. ChemSusChem.

[57] Wang Z., Zhou W., Wang X., Zhang X., Chen H., Hu H., Liu L., Ye J., Wang D. (2020). Enhanced Photocatalytic CO_2_ Reduction over TiO_2_ Using Metalloporphyrin as the Cocatalyst. Catalysts.

[58] Wu Y., Liu J.-C., Li R.-Z., Ci C.-G. (2022). Different Metal Upper Porphyrin Based Self-Assembly Sensitizers for Application in Efficient Dye-Sensitized Solar Cells. Polyhedron.

[59] Zhang J.-X., Wu Y., Liu J.-C., Li R.-Z. (2016). Bilayer Structured Supramolecular Light Harvesting Arrays Based on Zinc Porphyrin Coordination Polymers for Enhanced Photocurrent Generation in Dye Sensitized Solar Cells. Dalton Trans..

[60] Gong Y.-N., Zhong W., Li Y., Qiu Y., Zheng L., Jiang J., Jiang H.-L. (2020). Regulating Photocatalysis by Spin-State Manipulation of Cobalt in Covalent Organic Frameworks. J. Am. Chem. Soc..

[61] Hussain H., Tocci G., Woolcot T., Torrelles X., Pang C.L., Humphrey D.S., Yim C.M., Grinter D.C., Cabailh G., Bikondoa O. (2017). Structure of a Model TiO_2_ Photocatalytic Interface. Nat. Mater..

[62] Zhang L., Cole J.M. (2015). Anchoring Groups for Dye-Sensitized Solar Cells. ACS Appl. Mater. Interfaces.

[63] Kathiravan A., Renganathan R. (2009). Effect of Anchoring Group on the Photosensitization of Colloidal TiO_2_ Nanoparticles with Porphyrins. J. Colloid Interface Sci..

[64] Calmeiro J.M.D., Gira G., Ferraz F.M., Fernandes S.R.G., Pinto A.L., Lourenço L.M.O., Tomé J.P.C., Pereira C.C.L. (2020). Influence of the Meso-Substituents of Zinc Porphyrins in Dye-Sensitized Solar Cell Efficiency with Improved Performance under Short Periods of White Light Illumination. Dye. Pigment..

[65] Milana P., Nurhayati, Steky F.V., Ando Y., Simanullang M., Sugiyama M., Radiman C.L., Suendo V. (2020). Effect of Anchoring Groups on Electron Transfer at Porphyrins-TiO_2_ Interfaces in Dye-Sensitized Solar Cell Application. Macromol. Symp..

[66] Daphnomili D., Landrou G., Prakash Singh S., Thomas A., Yesudas K., Bhanuprakash K., Sharma G.D., Coutsolelos A.G. (2012). Photophysical, Electrochemical and Photovoltaic Properties of Dye Sensitized Solar Cells Using a Series of Pyridyl Functionalized Porphyrin Dyes. RSC Adv..

[67] Angaridis P.A., Ferentinos E., Charalambidis G., Ladomenou K., Nikolaou V., Biswas S., Sharma G.D., Coutsolelos A.G. (2016). Pyridyl vs. Bipyridyl Anchoring Groups of Porphyrin Sensitizers for Dye Sensitized Solar Cells. RSC Adv..

[68] Mai C.L., Moehl T., Hsieh C.H., Décoppet J.D., Zakeeruddin S.M., Grätzel M., Yeh C.Y. (2015). Porphyrin Sensitizers Bearing a Pyridine-Type Anchoring Group for Dye-Sensitized Solar Cells. ACS Appl. Mater. Interfaces.

[69] Brennan B.J., Llansola Portolés M.J., Liddell P.A., Moore T.A., Moore A.L., Gust D. (2013). Comparison of Silatrane, Phosphonic Acid, and Carboxylic Acid Functional Groups for Attachment of Porphyrin Sensitizers to TiO_2_ in Photoelectrochemical Cells. Phys. Chem. Chem. Phys..

[70] Zietz B., Gabrielsson E., Johansson V., El-Zohry A.M., Sun L., Kloo L. (2014). Photoisomerization of the Cyanoacrylic Acid Acceptor Group-a Potential Problem for Organic Dyes in Solar Cells. Phys. Chem. Chem. Phys..

[71] Martini L.A., Moore G.F., Milot R.L., Cai L.Z., Sheehan S.W., Schmuttenmaer C.A., Brudvig G.W., Crabtree R.H. (2013). Modular Assembly of High-Potential Zinc Porphyrin Photosensitizers Attached to TiO_2_ with a Series of Anchoring Groups. J. Phys. Chem. C.

[72] Guerrero G., Alauzun J.G., Granier M., Laurencin D., Mutin P.H. (2013). Phosphonate Coupling Molecules for the Control of Surface/Interface Properties and the Synthesis of Nanomaterials. Dalton Trans..

[73] Kumar P.R., Shajan X.S., Mothi E.M. (2020). Pyridyl/Hydroxyphenyl versus Carboxyphenyl Anchoring Moieties in Zn—Thienyl Porphyrins for Dye Sensitized Solar Cells. Spectrochim. Acta Part A Mol. Biomol. Spectrosc..

[74] Adineh M., Tahay P., Ameri M., Safari N., Mohajerani E. (2016). Fabrication and Analysis of Dye-Sensitized Solar Cells (DSSCs) Using Porphyrin Dyes with Catechol Anchoring Groups. RSC Adv..

[75] Fujimoto J., Hayashi S., Kainuma H., Manseki K., Udagawa T., Miyaji H. (2019). Supramolecular Light-Harvesting Antennas of Metal-Coordinated Bis(8-Hydroxyquinoline)-Substituted Porphyrin Networks. Chem. Asian J..

[76] Higashino T., Fujimori Y., Sugiura K., Tsuji Y., Ito S., Imahori H. (2015). Tropolone as a High-Performance Robust Anchoring Group for Dye-Sensitized Solar Cells. Angew. Chem. Int. Ed..

[77] Higashino T., Kurumisawa Y., Cai N., Fujimori Y., Tsuji Y., Nimura S., Packwood D.M., Park J., Imahori H. (2017). A Hydroxamic Acid Anchoring Group for Durable Dye-Sensitized Solar Cells Incorporating a Cobalt Redox Shuttle. ChemSusChem.

[78] Gou F., Jiang X., Fang R., Jing H., Zhu Z. (2014). Strategy to Improve Photovoltaic Performance of DSSC Sensitized by Zinc Prophyrin Using Salicylic Acid as a Tridentate Anchoring Group. ACS Appl. Mater. Interfaces.

[79] Hart A.S., KC C.B., Gobeze H.B., Sequeira L.R., D’Souza F. (2013). Porphyrin-Sensitized Solar Cells: Effect of Carboxyl Anchor Group Orientation on the Cell Performance. ACS Appl. Mater. Interfaces.

[80] Ambre R.B., Mane S.B., Chang G.-F., Hung C.-H. (2015). Effects of Number and Position of Meta and Para Carboxyphenyl Groups of Zinc Porphyrins in Dye-Sensitized Solar Cells: Structure–Performance Relationship. ACS Appl. Mater. Interfaces.

[81] Keawin T., Tarsang R., Sirithip K., Prachumrak N., Sudyoadsuk T., Namuangruk S., Roncali J., Kungwan N., Promarak V., Jungsuttiwong S. (2017). Anchoring Number-Performance Relationship of Zinc-Porphyrin Sensitizers for Dye-Sensitized Solar Cells: A Combined Experimental and Theoretical Study. Dye. Pigment..

[82] Tasseroul L., Páez C.A., Lambert S.D., Eskenazi D., Heinrichs B. (2016). Photocatalytic Decomposition of Hydrogen Peroxide over Nanoparticles of TiO_2_ and Ni(II)-Porphyrin-Doped TiO_2_: A Relationship between Activity and Porphyrin Anchoring Mode. Appl. Catal. B Environ..

[83] Safaei E., Mohebbi S. (2016). Photocatalytic Activity of Nanohybrid Co-TCPP@TiO_2_/WO_3_ in Aerobic Oxidation of Alcohols under Visible Light. J. Mater. Chem. A.

[84] Zeininger L., Lodermeyer F., Costa R.D., Guldi D.M., Hirsch A. (2016). Hydrogen Bonding Mediated Orthogonal and Reversible Self-Assembly of Porphyrin Sensitizers onto TiO_2_ Nanoparticles. Chem. Commun..

[85] Mathew S., Yella A., Gao P., Humphry-Baker R., Curchod B.F.E., Ashari-Astani N., Tavernelli I., Rothlisberger U., Nazeeruddin M.K., Grätzel M. (2014). Dye-Sensitized Solar Cells with 13% Efficiency Achieved through the Molecular Engineering of Porphyrin Sensitizers. Nat. Chem..

[86] Krishna N.V., Krishna J.V.S., Singh S.P., Giribabu L., Han L., Bedja I., Gupta R.K., Islam A. (2017). Donor-π–Acceptor Based Stable Porphyrin Sensitizers for Dye-Sensitized Solar Cells: Effect of π-Conjugated Spacers. J. Phys. Chem. C.

[87] Lu J., Li H., Liu S., Chang Y.-C., Wu H.-P., Cheng Y., Wei-Guang Diau E., Wang M. (2016). Novel Porphyrin-Preparation, Characterization, and Applications in Solar Energy Conversion. Phys. Chem. Chem. Phys..

[88] Chitpakdee C., Namuangruk S., Suttisintong K., Jungsuttiwong S., Keawin T., Sudyoadsuk T., Sirithip K., Promarak V., Kungwan N. (2015). Effects of π-Linker, Anchoring Group and Capped Carbazole at Meso-Substituted Zinc-Porphyrins on Conversion Efficiency of DSSCs. Dye. Pigment..

[89] Jinadasa R.G.W., Li B., Schmitz B., Kumar S., Hu Y., Kerr L., Wang H. (2016). Monobenzoporphyrins as Sensitizers for Dye-Sensitized Solar Cells: Observation of Significant Spacer-Group Effect. ChemSusChem.

[90] Ngo K.T., Rochford J., Fan H., Batarseh A., Chitre K., Rangan S., Bartynski R.A., Galoppini E. (2015). Photoelectrochemical Properties of Porphyrin Dyes with a Molecular Dipole in the Linker. Faraday Discuss..

[91] Panagiotakis S., Giannoudis E., Charisiadis A., Paravatou R., Lazaridi M.-E., Kandyli M., Ladomenou K., Angaridis P.A., Bertrand H.C., Sharma G.D. (2018). Increased Efficiency of Dye-Sensitized Solar Cells by Incorporation of a π Spacer in Donor–Acceptor Zinc Porphyrins Bearing Cyanoacrylic Acid as an Anchoring Group. Eur. J. Inorg. Chem..

[92] Ji J.-M., Kim S.H., Zhou H., Kim C.H., Kim H.K. (2019). D−π–A-Structured Porphyrins with Extended Auxiliary π-Spacers for Highly Efficient Dye-Sensitized Solar Cells. ACS Appl. Mater. Interfaces.

[93] Song H., Liu Q., Xie Y. (2018). Porphyrin-Sensitized Solar Cells: Systematic Molecular Optimization, Coadsorption and Cosensitization. Chem. Commun..

[94] Koposova E., Liu X., Pendin A., Thiele B., Shumilova G., Ermolenko Y., Offenhäusser A., Mourzina Y. (2016). Influence of Meso-Substitution of the Porphyrin Ring on Enhanced Hydrogen Evolution in a Photochemical System. J. Phys. Chem. C.

[95] Zhou H., Ji J.-M., Kim H.K. (2021). Porphyrin Sensitizers with Acceptor Structural Engineering for Dye-Sensitized Solar Cells. Dye. Pigment..

[96] Lu F., Zhang J., Zhou Y., Zhao Y., Zhang B., Feng Y. (2016). Novel D–π–A Porphyrin Dyes with Different Alkoxy Chains for Use in Dye-Sensitized Solar Cells. Dye. Pigment..

[97] Higashino T., Kawamoto K., Sugiura K., Fujimori Y., Tsuji Y., Kurotobi K., Ito S., Imahori H. (2016). Effects of Bulky Substituents of Push–Pull Porphyrins on Photovoltaic Properties of Dye-Sensitized Solar Cells. ACS Appl. Mater. Interfaces.

[98] Tang Y., Wang Y., Yan Q., Zeng K., Tang W., Zhao S., Kong C., Xie Y. (2021). Optimization of Porphyrin Dyes with a Bulky Triphenylamine Donor for Developing Efficient Dye-Sensitized Solar Cells. Dye. Pigment..

[99] Li S., Zhang Y., Mei S., Kong X., Yang M., Hu Z., Wu W., He J., Tan H. (2021). A Molecular Engineering Strategy of Phenylamine-Based Zinc-Porphyrin Dyes for Dye-Sensitized Solar Cells: Synthesis, Characteristics, and Structure–Performance Relationships. ACS Appl. Energy Mater..

[100] Prakash K., Alsaleh A.Z., Neeraj, Rathi P., Sharma A., Sankar M., D’Souza F. (2019). Synthesis, Spectral, Electrochemical and Photovoltaic Studies of A_3_B Porphyrinic Dyes Having Peripheral Donors. ChemPhysChem.

[101] Hu Y., Alsaleh A., Trinh O., D’Souza F., Wang H. (2021). β-Functionalized Push–Pull Opp-Dibenzoporphyrins as Sensitizers for Dye-Sensitized Solar Cells: The Push Group Effect. J. Mater. Chem. A.

[102] Duvva N., Prasanthkumar S., Giribabu L. (2019). Influence of Strong Electron Donating Nature of Phenothiazine on A_3_B- Type Porphyrin Based Dye Sensitized Solar Cells. Sol. Energy.

[103] Sirithip K., Prachumrak N., Rattanawan R., Keawin T., Sudyoadsuk T., Namuangruk S., Jungsuttiwong S., Promarak V. (2015). Zinc–Porphyrin Dyes with Different Meso-Aryl Substituents for Dye-Sensitized Solar Cells: Experimental and Theoretical Studies. Chem. Asian J..

[104] Liu Q., Sun Q., Gao W., Shen J., Zhang Y., Lu G., Chen Y., Yu S., Li X. (2022). Turning Built-in Electric Field of Porphyrin on Ti^3+^ Self-Doped Blue-TiO_2_ Hollow Nanospheres Boosts Peroxidase-like Activity for High-Performance Biosensing. Chem. Eng. J..

[105] Nikolaou V., Charalambidis G., Landrou G., Nikoloudakis E., Planchat A., Tsalameni R., Junghans K., Kahnt A., Odobel F., Coutsolelos A.G. (2021). Antenna Effect in BODIPY-(Zn)Porphyrin Entities Promotes H2 Evolution in Dye-Sensitized Photocatalytic Systems. ACS Appl. Energy Mater..

[106] Jiang J., Swierk J.R., Materna K.L., Hedström S., Lee S.H., Crabtree R.H., Schmuttenmaer C.A., Batista V.S., Brudvig G.W. (2016). High-Potential Porphyrins Supported on SnO_2_ and TiO_2_ Surfaces for Photoelectrochemical Applications. J. Phys. Chem. C.

[107] Orbelli Biroli A., Tessore F., Di Carlo G., Pizzotti M., Benazzi E., Gentile F., Berardi S., Bignozzi C.A., Argazzi R., Natali M. (2019). Fluorinated ZnII Porphyrins for Dye-Sensitized Aqueous Photoelectrosynthetic Cells. ACS Appl. Mater. Interfaces.

[108] Chmielewski P.J., Latos-Grażyński L. (2005). Core Modified Porphyrins—A Macrocyclic Platform for Organometallic Chemistry. Coord. Chem. Rev..

[109] Gupta I., Ravikanth M. (2006). Recent Developments in Heteroporphyrins and Their Analogues. Coord. Chem. Rev..

[110] Chatterjee T., Shetti V.S., Sharma R., Ravikanth M. (2017). Heteroatom-Containing Porphyrin Analogues. Chem. Rev..

[111] Sang Y., Liu H., Umar A. (2015). Photocatalysis from UV/Vis to Near-Infrared Light: Towards Full Solar-Light Spectrum Activity. ChemCatChem.

[112] Arnold L., Müllen K. (2011). Modifying the Porphyrin Core—A Chemist’s Jigsaw. J. Porphyr. Phthalocyanines.

[113] Lee J., Papatzimas J.W., Bromby A.D., Gorobets E., Derksen D.J. (2016). Thiaporphyrin-Mediated Photocatalysis Using Red Light. RSC Adv..

[114] Xie Y., Joshi P., Ropp M., Galipeau D., Zhang L., Fong H., You Y., Qiao Q. (2009). Structural Effects of Core-Modified Porphyrins in Dye-Sensitized Solar Cells. J. Porphyr. Phthalocyanines.

[115] Mane S.B., Hung C.-H. (2014). Synthesis of Carboxylate Functionalized A_3_B and A_2_B_2_ Thiaporphyrins and Their Application in Dye-Sensitized Solar Cells. New J. Chem..

[116] Mane S.B., Luo L., Chang G.-F., Diau E.W.-G., Hung C.-H. (2014). Effects of Core-Modification on Porphyrin Sensitizers to the Efficiencies of Dye-Sensitized Solar Cells. J. Chin. Chem. Soc..

[117] Mane S.B., Hu J.-Y., Chang Y.-C., Luo L., Diau E.W.-G., Hung C.-H. (2013). Novel Expanded Porphyrin Sensitized Solar Cells Using Boryl Oxasmaragdyrin as the Sensitizer. Chem. Commun..

[118] Vijay D., Varathan E., Subramanian V. (2013). Theoretical Design of Core Modified (Oxa and Thia) Porphyrin Based Organic Dyes with Bridging Thiophene Linkers. J. Mater. Chem. A.

[119] Misra R., Chandrashekar T.K. (2008). Structural Diversity in Expanded Porphyrins. Acc. Chem. Res..

[120] Osuka A., Saito S. (2011). Expanded Porphyrins and Aromaticity. Chem. Commun..

[121] Sung Y.M., Oh J., Cha W.-Y., Kim W., Lim J.M., Yoon M.-C., Kim D. (2017). Control and Switching of Aromaticity in Various All-Aza-Expanded Porphyrins: Spectroscopic and Theoretical Analyses. Chem. Rev..

[122] Woller T., Geerlings P., De Proft F., Champagne B., Alonso M. (2018). Aromaticity as a Guiding Concept for Spectroscopic Features and Nonlinear Optical Properties of Porphyrinoids. Molecules.

[123] Mori S., Kim K.S., Yoon Z.S., Noh S.B., Kim D., Osuka A. (2007). Peripheral Fabrications of a Bis-Gold(III) Complex of [26]Hexaphyrin(1.1.1.1.1.1) and Aromatic versus Antiaromatic Effect on Two-Photon Absorption Cross Section. J. Am. Chem. Soc..

[124] Yoon Z.S., Kwon J.H., Yoon M.-C., Koh M.K., Noh S.B., Sessler J.L., Lee J.T., Seidel D., Aguilar A., Shimizu S. (2006). Nonlinear Optical Properties and Excited-State Dynamics of Highly Symmetric Expanded Porphyrins. J. Am. Chem. Soc..

[125] Wu J.I., Fernández I., Schleyer P.v.R. (2013). Description of Aromaticity in Porphyrinoids. J. Am. Chem. Soc..

